# Fcmr regulates mononuclear phagocyte control of anti-tumor immunity

**DOI:** 10.1038/s41467-019-10619-w

**Published:** 2019-06-18

**Authors:** Shawn P. Kubli, Larsen Vornholz, Gordon Duncan, Wenjing Zhou, Parameswaran Ramachandran, Jerome Fortin, Maureen Cox, SeongJun Han, Robert Nechanitzky, Duygu Nechanitzky, Bryan E. Snow, Lisa Jones, Wanda Y. Li, Jillian Haight, Andrew Wakeham, Mark R. Bray, Tak W. Mak

**Affiliations:** 1 The Campbell Family Institute for Breast Cancer Research, Princess Margaret Cancer Centre, 610 University Avenue, Toronto, ON M5G 2M9 Canada; 2 Department of Medical Biophysics, University of Toronto, 101 College Street, Toronto, ON M5G 1L7 Canada; 3 Department of Immunology, University of Toronto, 101 College Street, Toronto, ON M5G 1L7 Canada; 4 Department of Medicine, University of Hong Kong, Pok Fu Lam, 999077 Hong Kong

**Keywords:** Immunology, Immunotherapy, Innate immune cells, Tumour immunology

## Abstract

Myeloid cells contribute to tumor progression, but how the constellation of receptors they express regulates their functions within the tumor microenvironment (TME) is unclear. We demonstrate that Fcmr (Toso), the putative receptor for soluble IgM, modulates myeloid cell responses to cancer. In a syngeneic melanoma model, Fcmr ablation in myeloid cells suppressed tumor growth and extended mouse survival. Fcmr deficiency increased myeloid cell population density in this malignancy and enhanced anti-tumor immunity. Single-cell RNA sequencing of Fcmr-deficient tumor-associated mononuclear phagocytes revealed a unique subset with enhanced antigen processing/presenting properties. Conversely, Fcmr activity negatively regulated the activation and migratory capacity of myeloid cells in vivo, and T cell activation by bone marrow-derived dendritic cells in vitro. Therapeutic targeting of Fcmr during oncogenesis decreased tumor growth when used as a single agent or in combination with anti-PD-1. Thus, Fcmr regulates myeloid cell activation within the TME and may be a potential therapeutic target.

## Abstract (web summary)

Myeloid cells modulate the immune response within the tumour microenvironment, but the underlying mechanisms remain largely unknown. Here, the authors show that Fcmr – the putative receptor for soluble IgM – is a potent negative regulator of anti-tumour immunity.

## Introduction

Immune cells have both positive and negative effects on tumor progression. Often these cell types work to eliminate neoplastic cells, but sometimes they in fact facilitate tumor growth by maintaining the tumor microenvironment (TME)^1,2^. Thus, while immunosurveillance protects against development of tumors, the infiltration into neoplastic growths of immunosuppressive mononuclear phagocytes (MPs) with trophic and tumor-promoting functions can facilitate malignant progression^2^. For many human cancers, the accumulation of MPs in the peripheral blood (PB) and within the tumor correlates with poor patient prognosis^2^. Accordingly, the depletion of MPs in various preclinical models has been shown to reduce both tumor growth^3–5^ and metastatic progression^6^.

While MPs can support tumor growth, they also promote anti-tumor immunity through antigen presentation and by orchestrating pro-inflammatory responses^1^. These dichotomous functions of MPs are largely dictated by the plethora of scavenger, co-stimulatory, and inhibitory receptors on their surface. The cross-talk among these receptors, through common, divergent, or antagonizing signaling pathways, affects numerous cellular functions, including phagocytosis, antigen presentation, and cytokine production^7,8^. Thus, gaining a clearer understanding of how various receptors modulate MP function within the TME is necessary for the identification of effective new immunotherapeutic targets for cancer treatment.

FCMR (Toso, Faim3), the putative receptor for soluble IgM, plays an important role in modulating immune responses during infection and autoimmunity (reviewed in ref. ^9^). FCMR has well-described cell type- and state-dependent functions in lymphocytes, including control of B cell receptor (BCR) signaling^10^, B2 cell development and function^9,11^, and Th17 cell production of inflammatory cytokines^12,13^. In addition, FCMR has cell-autonomous functions in both mouse and human myeloid cells^14–17^. *Fcmr* transcripts are expressed in mouse splenic neutrophils, dendritic cells (DCs), and to a lesser extent monocytes and macrophages (MΦ)^9,18^. Furthermore, cell-surface FCMR protein expression has been reported in bone marrow myeloid cells, including both bone marrow neutrophils and monocytes^14^. In addition, Fcmr expression can be induced in human MΦ upon exposure to modified lipids that activate scavenger receptors, and after complement-dependent phagocytosis^19^. *Fcmr* expression in MΦ and DCs has been identified in lung MΦ and CD103^+^ lung DCs in naive and orthotopic cancer setting^15^, adipose-associated MΦ^16^, and tissue repair-associated MΦ^17^. *Fcmr*^*−/−*^ mouse studies have provided some insights as to Fcmr acting within myeloid cells to facilitate clearance of bacterial and viral insults, promote cytokine production, and alter T cell responses^14,20^.

While Fcmr has been identified in various homeostatic and pathological conditions in myeloid cells, the functions of FCMR in these cells is not well defined. In particular, the potential influence of Fcmr on MP biology within the TME remains unexplored. FCMR expression in cell types that have important roles in modulating TME maintenance and anti-tumor immunity, such as monocytes, activated MΦ, and DCs, suggests a potential function for FCMR in myeloid cells function during cancer progression.

Based on Fcmr-dependent modulation of inflammatory and cell-mediated immune processes, which are also important in cancer, we hypothesized that Fcmr might play a role in modulating immune responses within the TME. Here we report that Fcmr acts within myeloid cells as a negative regulator of anti-tumor immunity. Mechanistically, Fcmr deficiency in myeloid cells leads to increased phagocytosis, enhanced antigen presentation, and heightened T cell activation. A Toso-Fc decoy receptor can reduce tumorigenesis in mice when used either as a single agent or in combination with anti-PD1 antibody. Our data suggest that therapeutic targeting of Fcmr may be a promising strategy for cancer treatment.

## Results

### Fcmr inhibits tumor immunity and is myeloid cell-dependent

**Fig. 1:**
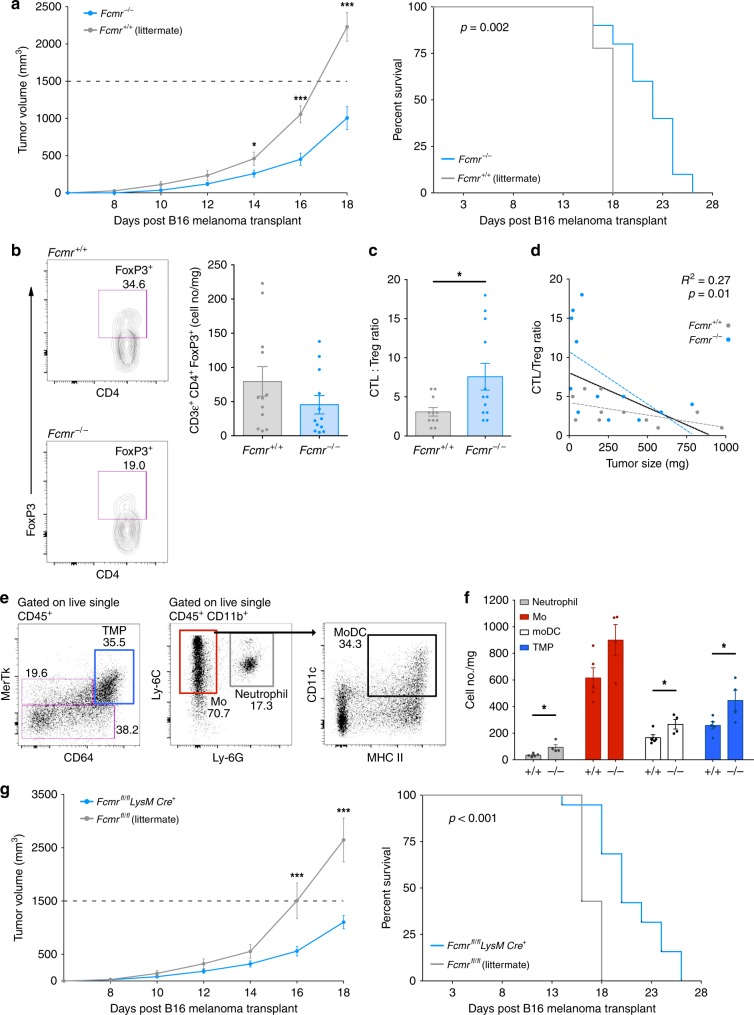
Fcmr inhibits myeloid cell-dependent anti-tumor immunity. **a** Tumor growth (left) and mouse survival (right) curves of *Fcmr*^*−/−*^ and *Fcmr*^*+/+*^ littermate mice that received ventral–lateral intradermal B16F0 cell transplants (2 × 10^5^ cells) at a site superior to the inguinal LN. Data are from one trial (*n* = 9 *Fcmr*^*+/+*^ and 8 *Fcmr*^*−/−*^ mice), and representative of 2 separate experiments. **b**–**d** CTL:Treg ratios in B16F0 tumors in the *Fcmr*^*−/−*^ and *Fcmr*^*+/+*^ mice in (**a**). **b** Left: Representative Treg flow cytometry data obtained from the analysis of B16F0 tumors harvested from *Fcmr*^*−/−*^ and *Fcmr*^*+/+*^ mice. Right: Quantification of the data in the left panel normalized to tumor mass. **c** CTL:Treg ratio calculated as the number of CD8α^+^ T cells per FoxP3^+^ CD4^+^ T cells. See Supplementary Fig. 1 for data summary and gating strategy. **d** Correlation of the CTL:Treg ratio in (**c**) with the tumor mass at time of analysis. Data are pooled from 2 separate experiments (total *n* = 11 *Fcmr*^*+/+*^ and 12 *Fcmr*^*−/−*^ mice). **e** Representative flow cytometry plots for intratumor myeloid cell populations, showing the gating strategy. **f** Quantification of the indicated cell subsets normalized to tumor mass (*n* = 5 *Fcmr*^*+/+*^ and 5 *Fcmr*^*−/−*^ mice). **g** Tumor growth (left) and mouse survival (right) curves of *Fcmr*^*fl/fl*^*;LysMCre*^*+*^ and *Fcmr*^*fl/fl*^ littermate mice that received B16F0 cells as described in (**a**). (*n* = 17 *Fcmr*^*fl/fl*^ and 7 *Fcmr*^*fl/fl*^; *LysMCre*^*+*^ mice). Data are represented as mean ± SEM (ANOVA, *t* test, linear regression; *p < 0.05; **p < 0.01; ***p < 0.001)

To determine whether Fcmr modulates immune responses during tumor development, we employed the B16 syngeneic melanoma cancer model. *Fcmr*^*−/−*^ mice receiving B16 transplants exhibited less aggressive tumor growth than their *Fcmr*^*+/+*^ littermates and showed prolonged survival (Fig. 1a, Supplementary Fig. 1a). Tumor-infiltrating lymphocyte (TIL) densities were similar between genotypes (Supplementary Fig. 1b, c), suggesting that delayed disease progression in *Fcmr*^*−/−*^ mice was not due to altered TIL access to the TME. Instead, fewer regulatory T cells (Treg) were found in tumors of *Fcmr*^*−/−*^ mice (Fig. 1b), and the ratio of cytotoxic T lymphocytes (CTL) to Treg was higher in tumors of *Fcmr*^*−/−*^ mice than in those of *Fcmr*^*+/+*^ mice (Fig. 1c). This CTL:Treg ratio correlated inversely with tumor weight at the time of analysis (Fig. 1d).

We were intrigued by the lack of difference in B cell density within B16 tumors in *Fcmr*^*+/+*^ and *Fcmr*^*−/−*^ mice (Supplementary Fig. 1c). To test whether Fcmr acts in B cells to regulate tumor growth, we transplanted B16 cells into mice in which Fcmr was ablated specifically in B cells (*Fcmr*^*fl/fl*^*;Mb1Cre*^*+*^). However, there were no differences in either B16 tumor growth or the survival of *Fcmr*^*fl/fl*^*;Mb1Cre*^*+*^ mice compared to their *Fcmr*^*fl/fl*^ littermates (Supplementary Fig. 1d). Therefore, loss of Fcmr’s functions in B cells does not explain the anti-cancer effect of global *Fcmr* deficiency.

Myeloid cells play a vital role in orchestrating anti-tumor immunity, and previous work indicates Fcmr expression in myeloid cells alters their function^14,15,18,19^. Interestingly, we found an increase in tumor-associated myeloid cell densities in *Fcmr*^*−/−*^ mice compared with their *Fcmr*^*+/+*^ littermates (Fig. 1e, f). Specifically, there was an approximately 2-fold increase in neutrophils (CD11b^+^ Ly-6C^int^ Ly-6G^+^), monocytic dendritic cells (MoDC, CD11b^+^ Ly-6C^+^ Ly-6G^−^ CD11c^+^ MHC II^+/hi^), and mature MPs (CD11b^+^ MerTk^+^ CD64^+^) in B16 tumors growing in *Fcmr*^*−/−*^ mice (Fig. 1f). Therefore, we generated *Fcmr*^*fl/fl*^*;LysMCre*^*+*^ mice in which *Fcmr* was selectively deleted in myeloid cells. Compared to their *Fcmr*^*fl/fl*^ littermates, *Fcmr*^*fl/fl*^*;LysMCre*^*+*^ mice exhibited a significant attenuation of tumor growth and experienced extended survival (Fig. 1g). Taken together, these findings suggest a myeloid cell-autonomous role for Fcmr in regulating tumor growth.

### scRNA-seq identifies Fcmr-dependent heterogeneity in TMPs

The mononuclear phagocytic system (MPS) is characterized by functional diversity and plasticity^21^, including within the TME^22^. Tumor-associated mononuclear phagocytes (TMPs) within the TME come from various ontological origins. To determine how Fcmr deficiency might impact the functional heterogeneity of mature MPs within the TME, we performed single-cell RNA sequencing (scRNAseq) on MPs isolated from B16 tumors resected from *Fcmr*^*−/−*^ and *Fcmr*^*+/+*^ mice. To evaluate a homogenous population of MPs within the TME we chose to examine cells harboring expression of markers found in more mature, differentiated MPs. Immune profiling using classic MP markers CD11b and F4/80 revealed that two MP subpopulations commonly observed in normal tissues (CD11b^lo^ F4/80^hi^ and CD11b^hi^ F4/80^lo^) were not readily discernable in B16 tumors (Supplementary Fig. 2a). However, when antibody-stained B16 TMPs were first gated on cells expressing MP markers identified by the Immunological Genome Project (CD64 and MerTk)^23^, CD11b^lo^ F4/80^hi^ and CD11b^hi^ F4/80^lo^ subsets could be clearly identified (Supplementary Fig. 2a). Therefore, we then sorted MerTk^+^ CD64^+^ TMPs by FACS and subjected these cells to single-cell transcriptomic analyses using the Chromium 10X Genomics platform. We obtained RNAseq profiles from 6352 *Fcmr*^*+/+*^ and 7999 *Fcmr*^*−/−*^ TMPs.

**Fig. 2:**
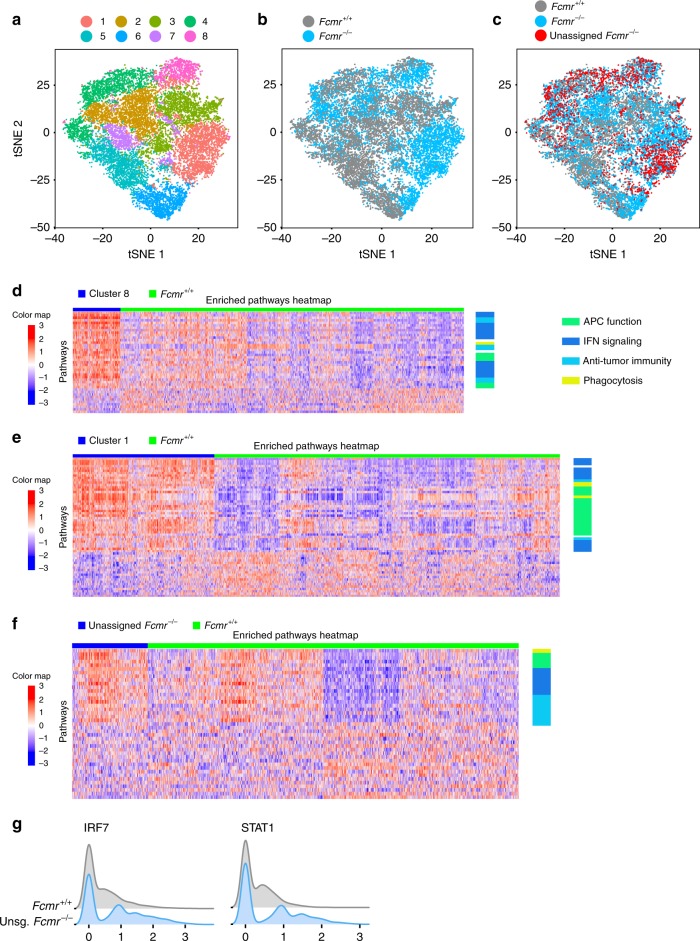
Fcmr deficiency alters TMP functional heterogeneity. **a** t-SNE representation of cell clusters from FACS-sorted tumor mononuclear phagocytes (TMP) isolated from B16 melanoma tumors growing in *Fcmr*^*+/+*^ and *Fcmr*^*−/−*^. **b** t-SNE representation of the FACS-sorted *Fcmr*^*+/+*^ and *Fcmr*^*−/−*^ TMPs in (**a**), highlighting the differential clustering of *Fcmr*^*+/+*^ (gray) and *Fcmr*^*−/−*^ (blue) cells. **c** t-SNE representation of the cells in (**b**) with an emphasis on the unassigned *Fcmr*^*−/−*^ cells (red) that were identified by a projection analysis of *Fcmr*^*−/−*^ cells onto *Fcmr*^*+/+*^ cell clusters. **d**–**f** Heat maps identifying signaling pathways that were significantly up- and down-regulated in various cell subsets as determined by gene set variation analysis (GSVA). Plotted values are the *z*-score normalized GSVA scores, with *z*-scores computed row wise. *Fcmr*^*+/+*^ cells were compared to either **d** cluster 8 from the combined analysis of *Fcmr*^*+/+*^ and *Fcmr*^*−/−*^ cells; **e** cluster 1 from the combined analysis of *Fcmr*^*+/+*^ and *Fcmr*^*−/−*^ cells; or **f** the unassigned *Fcmr*^*−/−*^ cells from the projection analysis. For (**d**–**f**), each row represents a distinct signaling pathway, and each column represents a single cell. The color scale indicates the level of pathway enrichment. The columns are hierarchically clustered using a Pearson correlation-based distance metric and the ward D2 agglomeration method. The rows are sorted in decreasing order of log2 fold changes or linearly-modeled GSVA scores, with the most upregulated pathway appearing at the top and the most downregulated pathway appearing at the bottom. See Supplementary Tables 1 and 2 for examples of differentially regulated pathways. **g** Ridge plots representing the expression levels of transcription factors that were significantly differentially expressed between unassigned (unsg) *Fcmr*^*−/−*^ TMPs and all *Fcmr*^*+/+*^ cells

Using Seurat^24^, including t-distributed stochastic neighbor embedding (t-SNE), we identified 8 unique subsets of TMPs among our combined *Fcmr*^*+/+*^ and *Fcmr*^*−/−*^ populations (Fig. 2a). Fcmr deficiency drove cluster formation, as illustrated by the domination of clusters 1 and 8 by *Fcmr*^*−/−*^ cells (Fig. 2b, c). Other identified TMP subpopulations included a more even mixture of *Fcmr*^*+/+*^ and *Fcmr*^*−/−*^ cells, so we further analyzed *Fcmr*^*+/+*^ and *Fcmr*^*−/−*^ TMPs separately using the same approach (Supplementary Fig. 2b, c). This analysis showed that *Fcmr*^*+/+*^ and *Fcmr*^*−/−*^ cells were distributed in 7 and 8 distinct clusters, respectively, further indicating that the presence of Fcmr determines TMP stratification. To assess the extent to which *Fcmr*^*−/−*^ cells could be matched to their *Fcmr*^*+/+*^ counterparts, we employed the projection methodology^25^. Each *Fcmr*^*−/−*^ cell was projected onto the cell clusters formed by *Fcmr*^*+/+*^ cells using a centroid-based gene expression similarity metric. We identified 1297 *Fcmr*^*−/−*^ cells, “unassigned cells”, which were transcriptionally distinct and could not be classified into any *Fcmr*^*+/+*^ TMP subpopulation (Supplementary Fig. 2c).

To identify the cellular processes that underlay the observed transcriptomic differences between *Fcmr*^*+/+*^ and *Fcmr*^*−/−*^ TMPs, we performed a pathway gene set variation analysis (GSVA)^26^. For these analyses, we focused on the cell clusters 1 and 8 dominated by *Fcmr*^*−/−*^ cells when *Fcmr*^*+/+*^ and *Fcmr*^*−/−*^ cells were analyzed together (Fig. 2a), as well as on the unassigned *Fcmr*^*−/−*^ cells from the projection analysis (Fig. 2c, Supplementary Fig. 2d, e). These populations of cells were then analyzed in comparison to all *Fcmr*^*+/+*^ cells combined (Supplementary Fig. 2b, c). The predominant pathways that were significantly and most highly upregulated in clusters 1 and 8 were those of interferon (IFN) type I and II signaling, and pathways implicated in the positive regulation of anti-tumor immune responses (Fig. 2d, e). Consistent with the domination in clusters 1 and 8 of *Fcmr*^*−/−*^ TMPs, the unassigned *Fcmr*^*−/−*^ cells from the projection analysis shared enrichment of several of these same pathways (Fig. 2f; Supplementary Table 1). In comparison, the unassigned *Fcmr*^*−/−*^ cells had lower expression of genes associated with immunosuppression, such as insulin-like growth factor receptor (IGF-1) and Wnt signaling, and tryptophan metabolism (Supplementary Table 2).

The strong representation of pathways involved in the IFN response as revealed by GSVA suggested that loss of Fcmr may affect immune responses to nucleic acids. Furthermore, the unassigned *Fcmr*^*−/−*^ cells showed upregulation of cytosolic DNA-sensing and endosomal toll-like receptor (TLR) associated pathways (Fig. 2f, Supplementary Table 1). Accordingly, we found that two important effectors of the IFN response, namely “Signal Transducer and Activator of Transcription 1” (*Stat1*) and “Interferon Regulatory Factor 7” (*Irf7*), were the only transcription factors to be significantly upregulated in unassigned *Fcmr*^*−/−*^ cells compared to *Fcmr*^*+/+*^ cells (Fig. 2g). Interestingly, eight other transcription factors were differentially expressed in unassigned *Fcmr*^*−/−*^ cells (Supplementary Fig. 2f), illustrating the complexity of TMP transcriptional profiles. Together, these data suggest that Fcmr negatively regulates tumor-associated antigen uptake and presentation, which could in turn affect MP-dependent activation of anti-tumor immunity.

### Fcmr influences dendritic cell antigen uptake and maturation

**Fig. 3:**
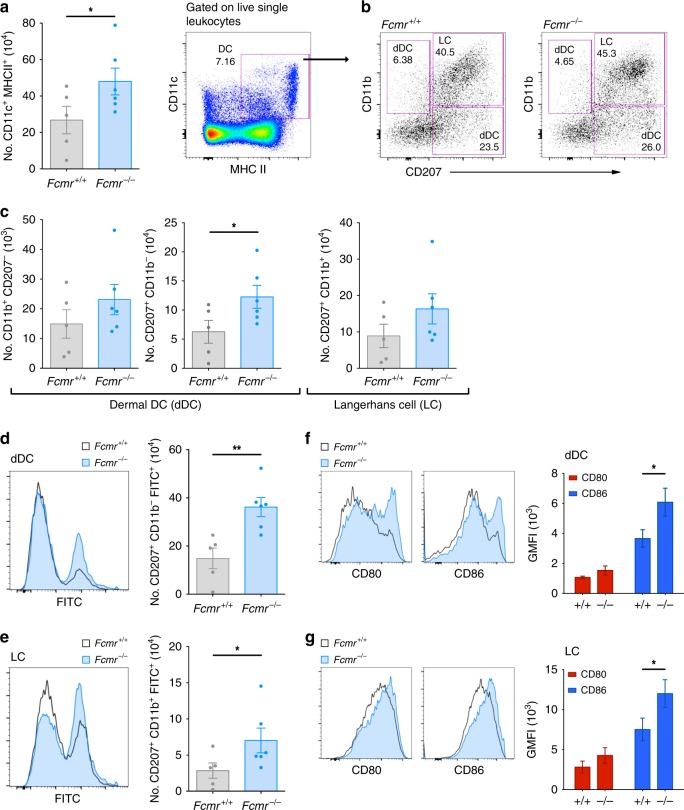
Fcmr inhibits skin DC migration, phagocytosis, and maturation. **a** Left: Quantification of total inguinal LN DCs in *Fcmr*^*+/+*^ and *Fcmr*^*−/−*^ mice at 24 h after ventral skin painting with FITC-dibutylthalate. Right: Representative flow cytometry plot showing the gating strategy. Data are representative of *n* = 5 *Fcmr*^*+/+*^ and 6 *Fcmr*^*−/−*^ mice. **b** Representative flow cytometry plots illustrating the gating strategy to identify the CD11b^+^ and CD11b^*−*^ subpopulations of dermal DCs (dDC) as well as Langerhans cells (LC) within the total DC population in the inguinal LN of the *Fcmr*^*+/+*^ and *Fcmr*^*−/−*^ mice painted with FITC-dibutylthalate in (**a**). **c** Quantification of the flow cytometry data collected on the two dermal DC and LC populations in (**b**). Each data point represents an individual mouse (*n* = 5 *Fcmr*^*+/+*^ and 6 *Fcmr*^*−/−*^ mice). **d**, **e** Left: Representative histograms showing FITC-negative and -positive subsets of the **d** dDC and **e** LC populations in the inguinal LN of the *Fcmr*^*+/+*^ and *Fcmr*^*−/−*^ mice in (**a**–**c**). Right: Quantification of the number of FITC-positive **d** dDC and **e** LC in the left panels. **f**, **g** Left: Representative histograms depicting cell surface levels of CD80 and CD86 on **f** dDC and **g** LC in the inguinal LN of the *Fcmr*^*+/+*^ and *Fcmr*^*−/−*^ mice in (**a–e**). Right: GMFI quantification of CD80 and CD86 expression levels on the **f** dDC and **g** LC in the left panel. Data are represented as mean ± SEM (*t* test; *p < 0.05; **p < 0.01; ***p < 0.001)

Based on our scRNAseq data, we hypothesized that increased expression of genes related to phagocytosis and antigen presentation/cross-presentation in Fcmr-deficient TMPs could explain the enhanced anti-cancer immunity in *Fcmr*^*−/−*^ mice. In the anatomical context of melanoma, this enhancement might reflect altered function of skin DCs. To explore this possibility, we first tested whether Fcmr intrinsically modulates the function of skin DCs. We painted the skin of *Fcmr*^*+/+*^ and *Fcmr*^*−/−*^ mice with FITC-dibutylthalate, a sensitizing agent that activates DCs to engulf FITC particles in the skin and subsequently migrate to the draining lymph node (LN)^27^ (Supplementary Fig. 3). In painted mice, the number of DCs (CD11c^+^ MHC II^+^) within the inguinal LN was higher in *Fcmr*^*−/−*^ mice than in their *Fcmr*^*+/+*^ littermates (Fig. 3a). After stratifying skin DC populations by lineage^28^, the greater number of DCs in *Fcmr*^*−/−*^ inguinal LN was found to reflect a significant increase in CD11b^−^ dermal DCs (dDCs; CD11c^+^ MHC II^+^ CD11b^−^ CD207^+^) as well as a trend towards higher numbers of Langerhans cells (LCs; CD11c^+^ MHC II^+^ CD11b^+^ CD207^+^) and CD11b^+^ dDCs (CD11c^+^ MHC II^+^ CD11b^+^ CD207^−^) (Fig. 3b, c). This observed increase in DCs in the absence of Fcmr suggests that Fcmr restricts the migration of activated skin DCs. Accordingly, numbers of FITC^+^ dDCs and LCs, as well as FITC geometric mean fluorescence intensity (GMFI), were elevated in *Fcmr*^*−/−*^ mice compared with their *Fcmr*^*+/+*^ littermates (Fig. 3d, e). FITC^+^ DCs in *Fcmr*^*−/−*^ mice also showed greater cell surface expression of the co-stimulatory molecules CD80 and CD86 (Fig. 3f, g). These data indicate that Fcmr negatively regulates DC maturation and function, and so might also affect TMP-mediated phagocytosis in the cancer setting.

### Fcmr suppresses MP uptake of cancer cell DNA

We predicted that the increased migration, phagocytosis, and upregulation of co-stimulatory molecules by DCs activated in *Fcmr*^*−/−*^ mice would be reflected in improved acute responses to B16 melanoma cells by skin DCs at the site of transplant. Previous work had shown that antigen-presenting cells (APCs) actively acquire cancer cell DNA. The DNA-sensing machinery of these APCs then bolsters their activation, priming T cell-mediated anti-tumor responses^29^.

**Fig. 4:**
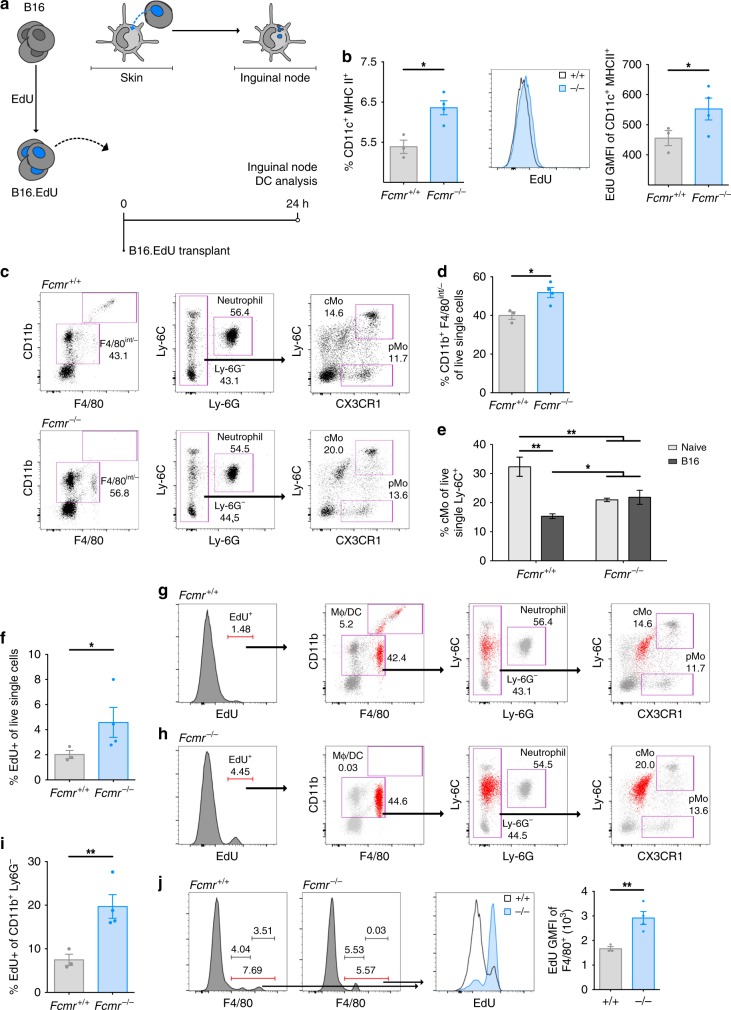
Fcmr inhibits phagocytosis to reduce cancer cell DNA uptake. **a** Schematic diagram showing B16 cell labeling with EdU (B16.EdU) (left), and their ventral–lateral transplantation via intradermal injection. The uptake of B16.EdU cells by skin DCs (top middle) and DC trafficking to the lymph node (top right) are also shown. The time course of the experiment is indicated (bottom right). **b** Left: Quantification of inguinal LN DCs in *Fcmr*^*+/+*^ and *Fcmr*^*−/−*^ mice at 24 h post-B16.EdU transplant. Middle: Representative histogram showing relative EdU-positivity of these cells from *Fcmr*^*+/+*^ and *Fcmr*^*−/−*^ mice. Right: GMFI quantification of EdU-positive DCs in inguinal LN. Each data point represents an individual mouse (*n* = 3 *Fcmr*^*+/+*^ and 4 *Fcmr*^*−/−*^ mice). **c** Flow cytometry plots illustrating the gating strategy to identify classical monocytes (cMo) and patrolling monocytes (pMo) in PB of the *Fcmr*^*+/+*^ and *Fcmr*^*−/−*^ mice in (**b**) at 24 h post-B16.EdU transplant. **d** Quantification of percentage of cMo (CD11b^+^ F4/80^int/*−*^) among PB myeloid cells from the mice in (**c**) at 24 h post-B16.EdU transplant. Each data point represents an individual mouse. **e** Quantification of percentage of cMo (CD11b^+^ F4/80^int/*−*^ Ly-6G^−^ Ly-6C^hi^ CX3CR1^+^) among PB myeloid cells from naive *Fcmr*^*+/+*^ and *Fcmr*^*−/−*^ mice (gray) compared to *Fcmr*^*+/+*^ and *Fcmr*^*−/−*^ mice at 24 h post-B16.EdU transplant (black). Data were collected and analyzed by flow cytometry as in (**c**). **f** Quantification of total EdU-positive single live cells in PB of the *Fcmr*^*+/+*^ and *Fcmr*^*−/−*^ mice in (**b**–**e**). **g**, **h** Representative flow cytometry plots back-gating (**g**) *Fcmr*^*+/+*^ and (**h**) *Fcmr*^*−/−*^ total EdU-positive cells to the same populations of PB myeloid cells illustrated in (**c**) at 24 h post-B16.EdU transplant. The back-gated EdU-positive population is illustrated in red. **i** Quantification of EdU-positive MPs (CD11b^+^ Ly6G^−^) in PB of the *Fcmr*^*+/+*^ and *Fcmr*^*−/−*^ mice in (**b–e**) at 24 h post-B16.EdU transplant. **j** Left: histograms illustrating total F4/80^int/+^ single live leukocytes in PB of *Fcmr*^*+/+*^ and *Fcmr*^*−/−*^ mice. Middle: histogram showing the EdU-positive and EdU-negative populations among the cells in the left panel. Right: GMFI quantification of the data in the middle panel. Data are represented as mean ± SEM (*t* test; *p < 0.05; **p < 0.01; ***p < 0.001)

To examine if Fcmr influences uptake of cancer cell DNA, we labeled B16 cells with the DNA dye EdU (B16.EdU) and intradermally transplanted these cells into *Fcmr*^*+/+*^ and *Fcmr*^*−/−*^ recipients. Inguinal LN were resected and analyzed 24 h later to assess skin DC uptake of cancer cell DNA and subsequent migration to the draining LN (Fig. 4a). DCs (CD11c^+^ MHC II^hi/+^), but not lymphocytes, in the LN of transplant recipients were EdU-positive, demonstrating that these DCs had taken up cancer cell DNA in the skin and migrated to the node (Supplementary Fig. 4a). The frequency of DCs in the node, as well as the level of EdU GMFI within these migratory DCs, was higher in *Fcmr*^*−/−*^ mice than in *Fcmr*^*+/+*^ mice (Fig. 4b). These observations suggested that Fcmr inhibits DC uptake of cancer cell DNA or other cellular debris in the skin, and may reduce DC migration to proximal draining LN. Interestingly, we also identified a small population of EdU^+^ monocytes in the spleen at 24 h post-B16.EdU cell transplant (Supplementary Fig. 4b).

While there are no reported abnormalities in monocyte or neutrophil frequencies in the bone marrow of naive *Fcmr*^*−/−*^ mice^14^, a complete characterization of PB monocytes in these animals has not been reported. Because we identified EdU^+^ monocytes in the spleen following B16.EdU cell transplant, we sought to characterize PB monocytes in naive and transplanted *Fcmr*^*+/+*^ and *Fcmr*^*−/−*^ mice. We found an increased frequency of patrolling monocytes (pMo; CD11b^+^ Ly-6G^−^ Ly-6C^*−*/lo^ CX3CR1^+/hi^) but a reduction in classical monocytes (cMo; CD11b^+^ Ly-6G^−^ Ly-6C^hi^ CX3CR1^+/hi^) in the PB of naive *Fcmr*^*−/−*^ mice compared to *Fcmr*^*+/+*^ littermates (Supplementary Fig. 4d). Transplant of B16.EdU cells led to the appearance of CD11b^hi^ F4/80^hi^ MPs in *Fcmr*^*+/+*^ mice but not in their *Fcmr*^*−/−*^ counterparts (Fig. 4c, Supplementary Fig. 4e). In B16.EdU cell transplant recipients, the percentage of CD11b^+^ F4/80^int/*−*^ myeloid cells among circulating PB cells was higher in *Fcmr*^*−/−*^ mice than in their *Fcmr*^*+/+*^ littermates (Fig. 4c, d). Unlike in *Fcmr*^*+/+*^ mice, B16.EdU cell transplantation in *Fcmr*^*−/−*^ mice did not reduce the relative representation of cMo, resulting in a higher proportion of these cells in these animals (Fig. 4e, Supplementary Fig. 4d). Together, these data suggest that the responses of PB MPs in *Fcmr*^*−/−*^ mice exposed to cancer cells are altered.

Consistent with our observations of draining node DCs, *Fcmr*^*−/−*^ mice had an increased frequency of total EdU^+^ cells in PB (Fig. 4f). Back-gating of EdU^+^ cells revealed two populations of EdU^+^ MPs in *Fcmr*^*+/+*^ mice but only one in their *Fcmr*^*−/−*^ littermates (Fig. 4g, h). Monocytic macrophage/DCs (MoMΦ/DC; CD11b^+^ F4/80^lo/int^ Ly-6G^−^ Ly-6C^int^ CX3CR1^int^ FSC-A^hi^; Supplementary Fig. 4d) were present in both *Fcmr*^*+/+*^ and *Fcmr*^*−/−*^ mice but at an increased ratio in *Fcmr*^*−/−*^ animals (Fig. 4d). However, *Fcmr*^*−/−*^ mice lacked the CD11b^hi^ F4/80^hi^ MP population observed in their *Fcmr*^*+/+*^ littermates (Supplementary Fig. 4e). Furthermore, the EdU GMFI value in total MPs was higher in *Fcmr*^*−/−*^ mice than in *Fcmr*^*+/+*^ mice (Fig. 4i, j). These results suggest that Fcmr influences the response of MPs to cancer cells by restraining their uptake of cancer cell DNA, and that this deficit impedes the maturation of these cells in the PB.

### Fcmr negatively regulates DC-dependent T cell activation

We next sought to delineate the mechanisms by which Fcmr reduces MP uptake of tumor-derived DNA and subsequent MP maturation, and examine if this has implications for T cell activation. For these investigations, we used bone marrow-derived DCs (BMDCs). MPs represent a heterogenous population of cells with different ontological origin and function. Macrophage (MΦ) and DCs comprise major, functionally distinct, populations of MPs. Subsets of MΦ and DCs have distinct roles in facilitating maintenance of tissue homeostasis and in responses to infection, autoimmunity, and cancer. While MΦ are APCs, DCs are specialized in performing this function and have increased capacity for phagocytosis, antigen presentation, and T cell activation. Based on the *Fcmr*^*−/−*^ transcriptional profile of TMPs that indicates heightened APC function (Fig. 2), and accompanying altered DC function in the skin of *Fcmr*^*−/−*^ mice (Fig. 3), we chose to perform functional studies using BMDCs, as this cell type is more specialized in antigen presentation than MΦ.

The stimulation of DNA-sensing pathways requires the engagement of particular pattern recognition receptors (PRRs), such as TLR-9 in endophagosomes and cyclic guanosine monophosphate-adenosine monophosphate synthase (cGAS) in the cytosol. Upon sensing CpG-rich unmethylated single-stranded (ss) DNA, TLR-9 signals to elements upstream of NFκB and IRF-7, and thereby modulates the activation of MPs that contribute to autoimmune and cancer pathologies^30^. Alternatively, cGAS detects cytosolic double-stranded (ds) DNA and promotes MP-dependent anti-tumor immunity^31^. Upon dsDNA binding, cGAS triggers a signaling cascade involving “stimulator of interferon genes” (STING) and “TANK-binding kinase 1” (TBK1) to activate NFκB and/or IRF-3 and expression of their target genes^32^, bolstering cancer cell killing.

**Fig. 5:**
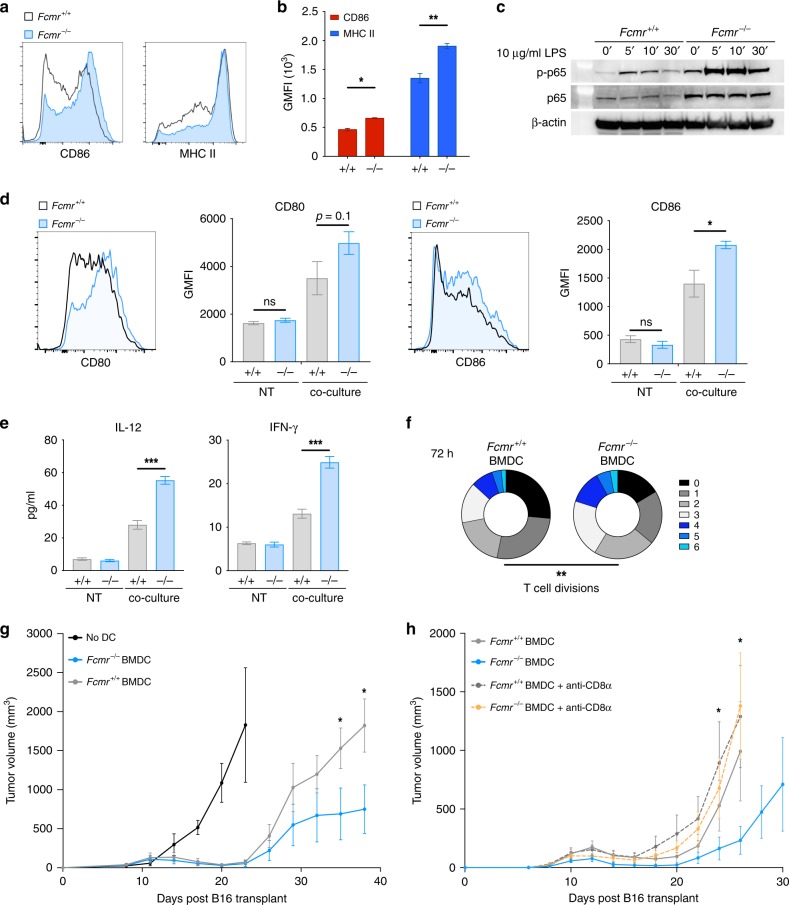
Fcmr inhibits DC maturation to impede T cell tumor immunity. **a** Representative histograms showing CD86 (left) and MHC II (right) expression by the CpG-stimulated *Fcmr*^*+/+*^ and *Fcmr*^*−/−*^ BMDCs. **b** Quantification of CD86 (red) and MHC II (blue) GMFI values from the *Fcmr*^*+/+*^ and *Fcmr*^*−/−*^ BMDCs. Data are pooled from three biological replicates. **c** Immunoblot analysis of total p65 and phospho-p65 protein levels in *Fcmr*^*+/+*^ (left) and *Fcmr*^*−/−*^ (right) BMDCs that were stimulated with 10 µg/ml LPS (to engage TLR-4) for the indicated times. β-actin, loading control. Data are representative of three separate biological replicates. **d** Left: Representative histogram of CD80 expression on *Fcmr*^*+/+*^ and *Fcmr*^*−/−*^ BMDCs that were co-cultured with moderately apoptotic B16 cells. Middle left: GMFI quantification of CD80 expression on *Fcmr*^*+/+*^ and *Fcmr*^*−/−*^ BMDCs that were left untreated (NT) or co-cultured with B16 cells. Middle right: Representative histogram of CD86 expression on these same BMDCs. Right: GMFI quantification of CD86 expression on BMDCs under NT or co-culture conditions. Bar graph data are pooled from three biological replicates. **e** Quantification of the indicated cytokines produced by *Fcmr*^*+/+*^ and *Fcmr*^*−/−*^ BMDCs under NT or co-culture conditions. Data are pooled from three biological replicates. **f** Quantification of T cell divisions at 72 h after co-culture with the *Fcmr*^*+/+*^ and *Fcmr*^*−/−*^ BMDCs. Data are from 3 technical replicates within each of 3 biological replicates. **g**, **h** Time course of tumor growth in *Fcmr*^*+/+*^ mice that received ventral–lateral intradermal transplant of B16gp33 cells (2 × 10^5^) superior to the inguinal LN and were vaccinated (*n* = 5 mice/group) with no BMDCs, or with *Fcmr*^*+/+*^ or *Fcmr*^*−/−*^ BMDCs as illustrated (Supplementary Fig. 7a). In a separate experiment, *Fcmr*^*+/+*^ tumor-burdened mice vaccinated with *Fcmr*^*+/+*^ or *Fcmr*^*−/−*^ BMDCs were treated with an anti-CD8α T cell depleting antibody as illustrated (Supplementary Fig. 7b). Data are represented as mean ± SEM (ANOVA, *t* test; *p < 0.05; **p < 0.01; ***p < 0.001)

To investigate Fcmr-dependent cell-autonomous differences in the activation of MPs by DNA, we stimulated bone marrow DCs (BMDCs) with either CpG or 2′-3′ cyclic guanosine monophosphate-adenosine monophosphate (cGAMP), the molecular intermediate produced by cGAS after dsDNA binding^32^. Control unstimulated *Fcmr*^*−/−*^ mice showed an approximately 2-fold increase in the frequency of mature BMDCs (CD11c^+^ MHC II^hi^) compared with their *Fcmr*^*+/+*^ littermates (Supplementary Fig. 5a). After stimulation of TLR-9 via CpG (Supplementary Fig. 5a), or STING via 2′3′-cGAMP (Supplementary Fig. 5b), *Fcmr*^*−/−*^ mice exhibited a further increase in mature BMDCs. This enhanced maturation of *Fcmr*^*−/−*^ BMDCs was reflected in their elevated expression of co-stimulatory molecules, including CD86 and MHC II (Fig. 5a, b; Supplementary Fig. 5c). Accordingly, compared with *Fcmr*^*+/+*^ BMDCs, *Fcmr*^*−/−*^ BMDCs exhibited heightened activation of the NFκB pathway following TLR stimulation, as evidenced by increases in both total p65 and p-p65 proteins (Fig. 5c). Together, these data suggest that *Fcmr*-deficient MPs undergo enhanced maturation upon PRR engagement.

Damage-associated molecular patterns (DAMPs) generated during cell death within necrotic regions of the TME provide an inflammatory stimulus leading to APC activation^1^. We therefore tested the response of *Fcmr*-deficient BMDCs to apoptotic/necrotic cancer cells. Thus, we co-cultured moderately apoptotic/necrotic B16 cells for 24 h with *Fcmr*^*−/−*^ or *Fcmr*^*+/+*^ BMDCs. The BMDCs were then examined for co-stimulatory receptor expression and inflammatory cytokine production. Corroborating our PRR stimulation experiments, *Fcmr*^*−/−*^ BMDCs from these co-cultures expressed higher levels of co-stimulatory molecules (particularly CD86) compared to *Fcmr*^*+/+*^ BMDCs (Fig. 5d). Both *Fcmr*^*+/+*^ and *Fcmr*^*−/−*^ BMDCs co-cultured with apoptotic/necrotic B16 cells also increased their production of the pro-inflammatory cytokines IL-12, IFN-γ, IL-1α, IL-1β, IL-6, and IL-10, as well as the growth factor GM-CSF (Fig. 5e; Supplementary Fig. 5d). However, the magnitude of this increase was substantially greater in *Fcmr*^*−/−*^ cultures than in the *Fcmr*^*+/+*^ controls. By contrast, the same co-culture conditions resulted in reduced production of IFN-β and TNF-α by BMDCs, but this effect was significantly blunted in *Fcmr*^*−/−*^ cultures (Supplementary Fig. 5d). These results suggest that Fcmr normally dampens MP activation in response to either PRR engagement or the complex stimulus of apoptotic/necrotic cells. Additionally, the loss of Fcmr results in increased co-stimulatory molecule expression and production of pro-inflammatory cytokines.

We reasoned, that by virtue of their enhanced activation phenotype upon co-culture with apoptotic/necrotic cells, *Fcmr*^*−/−*^ myeloid cells from the TME would stimulate T cell anti-tumor activity to an increased degree. To examine this, we first assessed the ability of *Fcmr*^*−/−*^ BMDCs to induce T cell proliferation in a T cell receptor (TCR)-dependent manner, using T cells that recognize antigen relevant to the melanoma cancer setting. *Fcmr*^*+/+*^ and *Fcmr*^*−/−*^ BMDCs were stimulated with CpG or cGAMP, pulsed with the melanocyte antigen Pmel peptide, and co-cultured with purified transgenic CD8α^+^ T cells expressing Pmel TCR that were stained with Cell Trace Violet (CTV). At 48 h after the initiation of co-culture, CD8α^+^ T cells co-cultured with *Fcmr*^*−/−*^ BMDCs had proliferated substantially more than those co-cultured with *Fcmr*^*+/+*^ BMDCs (Supplementary Figs. 5e and 6b). This difference in cell divisions was further exacerbated at 72 h after the initiation of co-culture (Fig. 5f; Supplementary Fig. 6c, d). Accordingly, the replication and proliferation indexes of CD8α^+^ T cells co-cultured with stimulated *Fcmr*^*−/−*^ BMDCs were greater than those of the same cells co-cultured with stimulated *Fcmr*^*+/+*^ BMDC for 72 h (Supplementary Fig. 6e, f). These observations suggest that Fcmr-deficient myeloid cells are more efficient at triggering melanocyte-specific T cell activation. Of note, previous work from myelin-oligodendrocyte glycoprotein (MOG)-specific T cells yielded different results^13^, suggesting that the antigen of interest and the model system influence *Fcmr*-dependent T cell activation.

We next used adoptive transfer to examine whether the enhanced activation of *Fcmr*^*−/−*^ DCs was associated with an intrinsic ability to induce tumor regression. Firstly, *Fcmr*^*+/+*^ mice were intradermally transplanted with B16 tumor cells expressing the gp33 peptide of lymphocytic choriomeningitis virus (LCMV) (B16gp33)^33^. On days 6 and 8 post-transplant, these mice received a contralateral intradermal vaccine of *Fcmr*^*+/+*^ or *Fcmr*^*−/−*^ BMDCs that had been stimulated in vitro with a TLR-9 agonist for 18 h, and pulsed with gp33 and gp100 peptides (Supplementary Fig. 7a). The intradermal DC injection route was selected based on previous evidence that cutaneous administration of DC vaccines is more efficacious^34^. We found that transfer of BMDCs from both genotypes resulted in tumor regression in recipient mice (Fig. 5g). However, soon after, the malignancies began to re-develop in both groups of animals (Fig. 5g). Strikingly, this tumor relapse was significantly less aggressive in mice receiving *Fcmr*^*−/−*^ BMDCs compared to those receiving *Fcmr*^*+/+*^ BMDCs (Fig. 5g).

To further clarify the involvement of CTL in the phenotypic reduction in tumor growth kinetics observed in mice receiving *Fcmr*^*−/−*^ BMDCs, we depleted CTLs after DC transfer. Again, we adoptively transferred *Fcmr*^*−/−*^ and *Fcmr*^*+/+*^ DCs to wildtype tumor-burdened mice (Supplementary Fig. 7b). Subsequently, we depleted CTLs via administration of an anti-CD8α monoclonal antibody (mAb) 12 and 14 days after B16 inoculation (Supplementary Fig. 7b). Administration of anti-CD8α mAb resulted in depletion of CD8α^+^ T cells (Supplementary Fig. 7c), but did not significantly alter tumorigenesis in mice receiving *Fcmr*^*+/+*^ DCs (Fig. 5h). However, in mice receiving *Fcmr*^*−/−*^ DCs, depletion of CD8α^+^ T cells resulted in accelerated tumor growth, that was similar to mice receiving *Fcmr*^*+/+*^ DCs. (Fig. 5h). Interestingly, 16 days after B16 inoculation we also observed increased antigen-experienced CD8α^+^ PD-1^+^ T cells in the PB of mice receiving *Fcmr*^*−/−*^ DCs (Supplementary Fig. 7d). Together, these data indicate that the adoptive transfer of *Fcmr*^*−/−*^ DCs promotes more robust anti-tumor responses, mediated through the activation of anti-tumor CD8α^+^ T cells. Collectively, these findings support our contention that the heightened activation of Fcmr-deficient DCs enhances T cell activation and results in improved anti-tumor immunity.

### Blockade of Fcmr cooperates with anti-PD-1 therapy

**Fig. 6:**
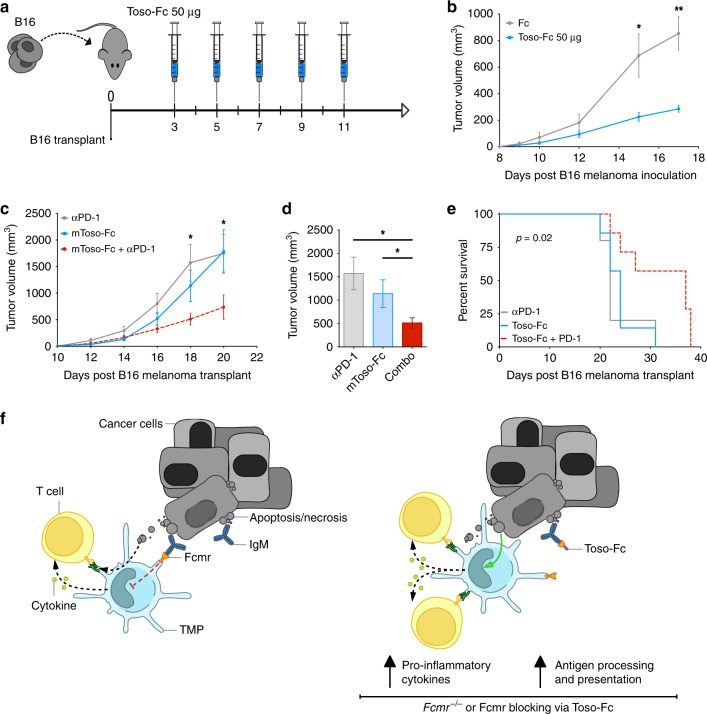
Toso-Fc reduces tumorigenesis when combined with anti-PD-1. **a** Schematic diagram of the experiment showing the ventral–lateral transplantation of B16F0 cells superior to the inguinal LN via intradermal injection into *Fcmr*^*+/+*^ recipient mice (left), and the associated timeline of treatment with Toso-Fc fusion protein decoy receptor starting 3 days post-transplant (right). **b** Time course of tumor growth in the *Fcmr*^*+/+*^ mice receiving Toso-Fc or Fc control treatment as illustrated in (**a**) (*n* = 10 mice/group). Data are representative of three separate experiments. **c** Time course of tumor growth in *Fcmr*^*+/+*^ mice transplanted as in (**a**) and receiving either anti-PD-1 antibody or Toso-Fc alone, or in combination, starting at 6 days post-B16 cell transplant (*n* = 8 mice/group). **d** Quantification of tumor volumes in the mice in (**c**) at 18 days post-B16 cell transplant. **e** Survival curves for the mice in (**c**). **f** Schematic diagram illustrating the functional implications of Fcmr ligation in MPs within the TME. Left: Fcmr–ligand interactions normally limit antigen processing/presentation and pro-inflammatory cytokine production by MPs, resulting in reduced T cell activation and decreased anti-tumor immune responses. Right: The Toso-Fc decoy receptor is hypothesized to out-compete Fcmr for binding to its ligand, preventing Fcmr–ligand interaction and thus removing the restraints on MP activation. The resulting increases in antigen processing/presentation and pro-inflammatory cytokine production promote T cell-mediated anti-tumor immunity. Toso-Fc is thus a potentially valuable immunotherapeutic agent. Data are represented as mean ± SEM (ANOVA *t* test; *p < 0.05; **p < 0.01; ***p < 0.001)

As genetic ablation of Fcmr enhanced MP activation and attenuated B16 tumor growth, we explored whether blockade of Fcmr–ligand interactions during cancer progression could have similar effects. We previously developed a Fc-fusion protein comprised of the extracellular region of Fcmr linked to the immunoglobulin heavy chain constant region (Fc), forming a decoy Fcmr receptor (Toso-Fc)^13^. We asked whether Toso-Fc, which blocks Fcmr activity, could recapitulate the anti-tumor effect of genetic *Fcmr* ablation. We transplanted *Fcmr*^*+/+*^ mice intradermally with B16 cells and treated them every other day (starting at 3 days post-transplant) with either 50 µg Toso-Fc or the stoichiometric equivalent of Fc control protein (Fig. 6a). Compared with mice receiving the Fc control protein, animals injected with Toso-Fc experienced a remarkable attenuation in tumor growth that was characterized by the appearance of significantly smaller tumors starting on day 15 after treatment (Fig. 6b), which corresponded with significantly increased survival (Supplementary Fig. 8a).

We observed that stimulating *Fcmr*^*−/−*^ BMDCs with 2′3′-cGAMP resulted in the upregulation of the T cell inhibitory receptor PD-L2 (Supplementary Fig. 5c), and that reduced tumor growth in *Fcmr*^*−/−*^ DC transfer recipients was dependent on CD8^+^ T cells (Fig. 5h). Therefore, we hypothesized that treatment of tumor-bearing mice with Toso-Fc in combination with PD-1 blockade might synergistically attenuate tumor growth. We administered Toso-Fc and anti-PD-1 antibody either individually or in combination (starting at 6 days post-transplant) to *Fcmr*^*+/+*^ mice transplanted with B16 tumor cells (Supplementary Fig. 8b). 18 days post-transplant, tumor sizes in mice receiving both Toso-Fc and anti-PD-1 treatments were much smaller than those developing in mice that received either treatment alone (Fig. 6c). Administration of either Toso-Fc or anti-PD-1 alone gave comparable beneficial results (Fig. 6c, d). The reduced tumor growth in mice receiving combination therapy significantly prolonged the survival of these animals (Fig. 6e). Thus, Fcmr inhibition may be a useful adjunct to existing immunotherapies.

## Discussion

Here, we provide the first evidence of a role for Fcmr in controlling immune responses within the TME. Our data reveal a myeloid cell-intrinsic role for Fcmr in down-regulating DC maturation and consequently T cell activation such that tumorigenesis is free to progress. Given previous studies implicating Fcmr in B cell development and function during infection^10,11,18^, our observation that conditional deletion of Fcmr in B lymphocytes did not affect B16 tumor cell growth was somewhat unexpected. However, Fcmr clearly modulates the development and function of various B cell populations, and so may affect the responses of B cells to tumors in situations where these cells contribute to disease pathogenesis^35^. Our examinations using the B16 melanoma model unequivocally demonstrate the critical influence of Fcmr within myeloid cells. Our single-cell transcriptomic analyses and functional experiments establish that Fcmr negatively regulates processes related to tumor antigen uptake and presentation by MPs. Our results support a model in which Fcmr ligation normally acts to limit DC maturation within the TME (Fig. 6f, left), providing another brake on anti-tumor T cell responses. Upon genetic ablation of *Fcmr* or blockade of FCMR–ligand interaction with a decoy receptor, this brake on DC activation is released, allowing vigorous uptake of tumor cell-derived antigens and cytokine production that promote T cell activation (Fig. 6f, right). Anti-tumor immunity is thus enhanced, and malignant cell growth is curtailed.

In addition to this newly described role in anti-tumor immunity, FCMR is also clearly critical for autoimmunity, and for immune responses to bacteria and virus. Thus, FCMR functions in B cells, T cells, and DCs, all of which play an important role during various immune responses. Clearly identifying which Fcmr-expressing cell types are impacting specific immune responses is of paramount importance to delineate FCMR’s functions. B cell-specific deletion of Fcmr suggests a B cell-dependent modulation of complex immune cell networks, which are important for the control and/or elimination of both bacterial and viral insults^9,11^. In addition, Fcmr also plays a clear T cell-intrinsic role in promoting pathogenic Th17 cell function. Furthermore, in the present manuscript, we clearly show that conditional deletion of Fcmr in B cells does not alter B16 melanoma tumor cell rejection. In contrast, we demonstrate that FCMR within the myeloid lineage significantly impacts tumor rejection. Through DC adoptive cell transfer experiments, we also show a DC-intrinsic effect of Fcmr during anti-tumor immune responses in vivo. Therefore, we suggest that different pathologies require FCMR function within different immune cell types. Differences in T cell priming observed in the various disease settings is likely explained by the immune cell networks driving pathogenesis. Fcmr expression in B cells influences the response to infection, Fcmr expression in T cells influences autoimmune responses, and Fcmr expression in myeloid cells regulates antitumor immunity in the B16 melanoma model.

*Fcmr*^*−/−*^ mice have reduced peripheral tolerance^9^. While this phenotype has been reported to be B cell-dependent, our results suggest a potential contribution of FCMR’s function in MPs as well. That is, the production of proinflammatory cytokines by MPs, which we demonstrate can be altered by FCMR ablation, may in turn influence B cell function, thus affecting immunity and tolerance. Specifically, we find that *Fcmr*^*−/−*^ MPs are more easily activated by apoptotic cells and produce more pro-inflammatory cytokine. In some contexts, increased cytokine production may positively influence B cell autoimmunity and partially explain the increased in autologous antibody production observed in full-body *Fcmr*^*−/−*^ mice^18,36,37^. Reciprocally, if soluble IgM is the sole ligand for FCMR, IgM antibody production by B cells could in turn modulate myeloid cell function via FCMR. This may partially explain previously identified alterations in myeloid cell activation in mice devoid of lymphocytes^38^. Ultimately, clearly identifying important autocrine and paracrine signals between various immune cells that express Fcmr will further clarify the impacts of this receptor’s expression on immune development and function.

An important aim in future investigations will be delineating the cellular mechanisms by which Fcmr dampens myeloid cell activation. Our data suggest that this effect is achieved at least partially by controlling the response of myeloid cells to nucleic acids. We showed that ablation of Fcmr resulted in enhanced TLR-dependent activation of its signaling effector NFκB, and our single-cell transcriptomic analyses revealed the upregulation of the transcription factors *Stat1* and *Irf7* in Fcmr-deficient cells within the TME. There is substantial cross-talk between the major transcriptional regulators NFκB and STAT. Specifically, TRAF6, a ubiquitin ligase acting upstream of NFκB activation, also ubiquitinates STAT and thereby negatively regulates JAK-STAT signaling^39^. Interestingly, Fcmr contains a putative TRAF2/6 binding motif^40^, providing a potential link between Fcmr engagement and alteration of STAT and NFκB activities. Furthermore, identification of Fcmr intracellular interactors should provide a better understanding of how this receptor regulates myeloid cell activation.

Our findings have particular relevance for cancer treatment by immunotherapy because our data demonstrate that interrupting Fcmr ligation in vivo can reduce tumor growth and prolong survival. Currently, FDA-approved immunotherapy-based treatments for melanoma (and other malignancies) involve blockade of PD-1, alone or in combination with anti-CTLA-4^41^. However, a substantial proportion of patients do not respond to this approach, highlighting the need for better treatments^41,42^. In this context, our experiments showing that blockade of Fcmr on myeloid cells synergizes with T cell-specific anti-PD1 treatment to reduce B16 melanoma growth suggest that therapeutic targeting of Fcmr may be a promising strategy for patients that do not respond to currently available immunotherapies. A better understanding of the signaling mechanisms that mediate the effects of Fcmr on myeloid cell activation may lead to the identification of additional therapeutic targets that can be exploited for cancer treatment.

## Methods

### Mice

*Fcmr*^*−*/*−*^^14^, *Fcmr*^fl/fl^ (KOMP Repository, University of California at Davis), MB1Cre^[+/wt 43^, LysMCre^[+/wt 44^, and *Pmel-1*^45,46^ mice were bred at the Princess Margaret Cancer Centre in the Ontario Cancer Institute under pathogen-free conditions. Sex-, age-, and cage-matched littermate control mice were used for all experiments. For therapeutic and DC vaccination experiments, 6–8-week-old C57Bl/6 female mice were purchased from Jackson Laboratories. For DC vaccination experiments, BMDC donor mice were *Fcmr*^*+/+*^ and *Fcmr*^*−/−*^ littermates, whereas tumor-bearing mice receiving BMDC vaccines were of the C57Bl/6 strain. Animal procedures were approved and performed in accordance with the animal care guidelines of the University Health Network Institutional Animal Care and Use Committee. Thus, all experiments were performed in compliance with the relevant ethical regulations for animal testing and research.

### B16 cell culture and transplantation

B16F0 melanoma cancer cells were obtained from ATCC and cultured in IMDM (Gibco) supplemented with 10% fetal bovine serum (FBS) (Seradigm), 2 mM L-glutamine (Gibco), 100 IU penicillin and 100 μg/ml streptomycin (Gibco). After culture initiation, B16F0 cells were passaged once and harvested using 0.05% Trypsin-EDTA (Gibco). Harvested cells were washed twice with 45 ml phenol-red free (PRF) HBSS and resuspended at 4 × 10^6^ cells/ml in HBSS. For transplants, mice received a ventral–lateral intradermal injection superior to the inguinal LN of 2 × 10^5^ viable B16F0 cancer cells in a volume of 50 μl. For labeling experiments, B16F0 cells were labeled for 2 h at 37 °C with 10 μM EdU (B16.EdU cells) prior to harvest. Viable B16.EdU cells (1 × 10^6^) in a 100 μl volume were then transplanted into recipients superior to the inguinal LN as above.

### Isolation of tumor-infiltrating cells

Tumor-bearing mice were euthanized in accordance with animal use protocol guidelines. Tumors ranging in size from 500 to 1000 mm^3^ were resected and weighed to the nearest 0.001 g, diced into 1–2 mm^3^ pieces, and subjected to enzymatic digestion with a Tumor Dissociation Kit (Miltenyi Biotec) following the manufacturer’s protocol. Dissociation was performed in C-tubes (Miltenyi Biotec) at 37 °C on a GentleMACS Octo-MACS Dissociator with Heaters (Miltenyi, Biotec) prior to immune cell isolation and analysis via flow cytometry or fluorescence-activated cell sorting (FACS) (see below).

### Flow cytometry and FACS

For antibody staining of cell surface molecules, isolated cells were passed through a 40 µm nylon mesh, aliquoted into 5 ml polystyrene FACS tubes, and washed with 2 ml PBS supplemented with 1% bovine serum albumin (BSA) plus 2 mM EDTA (referred to as FB buffer). Cells were pelleted at 300 g for 5 min at 4 °C, resuspended in 100 μl FB, and incubated for 10 min at 4 °C with 75 μl 1:200 Fc Block (αCD16/32 2.4G2, Tonbo Bioscience; rat serum, StemCell) in FB containing DNase I (Roche). Concentrated surface staining antibody cocktail (see below) (8×; 25 μl) was added to a total volume of 200 μl and incubation continued for 30 min at 4 °C. Stained cells were washed by centrifugation in 2.5 ml FB at 300*g* for 5 min at 4 °C. Washed cells were resuspended in 12.5 ng/ml DAPI (Sigma), and analyzed by flow cytometry (Fortessa, BD Biosciences) or sorted by FACS (FACSAriaII, BD Biosciences) as above.

For transcription factor detection, aliquoted cells were washed in 2 ml PBS by centrifugation at 300*g* for 5 min at 4 °C, followed by resuspension of the pellets and staining with Fixable Viability Dye eFluor 455 (UV) (eBioscience) according to the manufacturer’s instructions. Stained cells were washed with FB and immunostained to detect surface molecules as above. Immunostained cells were washed in 2.5 ml PBS by centrifugation at 300*g* for 5 min at 4 °C, resuspended in 250 μl FoxP3 Fixation/Permeabilization reagent (eBioscience), and incubated at 4 °C for either 1 h or overnight. Cells were washed in 1 ml Permeabilization Buffer (PeB) by centrifugation at 400*g* for 5 min at 4 °C. Antibodies against transcription factors were added at a 1:100 dilution, and the cells incubated for 30–60 min at 4 °C. Cells were washed twice with 2 ml PeB and resuspended in FB for flow cytometric analysis (Fortessa, BD Biosciences).

The following antibodies were used in this study: CD45.2-Alexa Fluor 700 (1:200, BioLegend, 109821), CD3ε-PE clone 145-2C11 (1:100, BioLegend, 100307), CD3ε-FITC clone 17A2 (1:100, BioLegend, 100203), CD4-PE-Cy7 clone RM4–5 (1:200, BioLegend, 100527), CD8-APC-Cy7 clone 53-6.7 (1:200, BioLegend, 100713), NK1.1-FITC clone PK136 (1:200, BioLegend, 108705), CD11b-Pacific Blue clone M1/70 (1:400, BioLegend, 101223), CD11c-APC and APC-Cy7 clone N418 (1:200, BioLegend, 117309 and 117323), CD64-FITC clone X54-5/7.1 (1:200, BioLegend, 139315), MHC-II(I-A/I-E)-PE-Cy7 clone M5/114.15.2 (1:2000, BioLegend, 107629), F4/80-PerCPCy5.5 and FITC clone BM8 (1:200, BioLegend, 123127 and 123107), L7-6C-brilliant violet 605 and PE-Cy7 clone HK1.4 (1:800, BioLegend, 128035 and 128017), Ly-6G-ApC-Cy7 clone 1A8 (1:200, BioLegend, 127623), CD80-PE and brilliant violet 605 clone 16-10A1 (1:400, BioLegend, 104707 and 104729), CD86-brilliant violet 421 and PE-Cy5 clone GL-1 (1:400, BioLegend, 105031 and 105015), CD273/PD-L2-APC clone TY25 (1:400, BioLegend, 107210), CX3CR1-PE clone SAO11F11 (1:200, BioLegend, 149005), CD192/CCR2-Alexa Fluor 647 clone SA203G11 (1:200, BioLegend, 150603), CD19-APC clone 1D3 (1:400, BD, 561738), CD40-PE clone 3/23 (1:400, BD, 553791), Zbtb-46-PE clone U4-1374 (1:50, BD, 565832), CD207-Alexa Fluor 647 clone 929F3.01 (1:200, Dendritics, DDX0362), MerTK-PE clone 108928 (1:100, R&D Systems, FAB5912P), FoxP3-APC clone FJK-16s (1:100, ThermoFisher, 17-5773-82).

### Single-cell sequencing and analysis

Tumor mononuclear phagocytes (TMPs) were isolated via FACS, washed, assessed for viability and morphology, and loaded onto a 10X Chromium instrument (10X Genomics) according to the manufacturer’s instructions. Single-cell RNA sequence libraries were prepared using the Chromium Single Cell 3′ Reagent Kit (10X Genomics) according to manufacturer’s instructions by the Princess Margaret Genomics Centre.

The raw sequencing reads were processed for quality control, mapping (to mouse genome build GRCm38), and UMI (unique molecular identifier) count matrix assembly using the CellRanger bioinformatics pipeline v2.0.1 provided by 10X Genomics. The assembled matrix of raw UMI counts, with 27,998 rows (genes) and 14,409 columns (cells), was then fed into the standard workflow of the single-cell data processing R package, Seurat v2.1.0^24^. Only genes that were expressed in at least 3 cells, and only cells that expressed at least 200 genes, were retained for downstream processing. Furthermore, cells expressing more than 7000 genes (potential multiplets), and cells with more than 30% of UMIs mapping to mitochondrial genes, were removed from the analysis. The final filtered matrix contained 16,112 genes and 14,351 cells, with 6352 *Fcmr*^+/+^ cells and 7999 *Fcmr*^*−*/*−*^ cells.

The filtered matrix was log-normalized using global scaling in Seurat with the scaling factor value set to 10,000. To identify highly variable genes, the *FindVariableGenes* module of Seurat was employed to establish the mean–variance relationship of the normalized counts of each gene across cells. Genes whose log-mean was between 0.0125 and 3 and whose dispersion was above 0.5, were chosen, resulting in 1339 highly varying genes. The normalized matrix was scaled and centered gene-wise, and then subjected to dimensionality reduction by carrying out principal component analysis (PCA) on the highly varying genes. Upon a visual inspection of the PCA elbow plot, which plots the standard deviations of the principal components (PCs), the top 10 PCs were chosen for further analysis.

Clustering was performed on the chosen PCs using the shared nearest neighbor (SNN) modularity optimization algorithm in Seurat, with default parameters. To visualize the clusters in two dimensions, a t-SNE map was computed and plotted using the *RunTSNE* and *TSNEPlot* modules of Seurat, respectively. Cluster-defining canonical marker genes were identified by comparing the gene-by-gene average expression levels within a cluster with the average levels across the rest of the cell population using the bimod likelihood ratio test for single cell gene expression^47^.

To determine whether, and to what extent, *Fcmr*^*−/−*^ cells could be matched to clusters formed exclusively by *Fcmr*^*+/+*^ cells, we employed the scmap R package^25^. This method projects cells of interest (query cells) one by one onto any reference set of clusters to identify the cluster to which the query cell is most similar. Projection was carried out by first computing the expression centroids (vectors of gene-wise median expression values) of the *Fcmr*^*+/+*^-exclusive clusters and then measuring similarity between the *Fcmr*^*−/−*^ cells and the centroids. Three similarity measures (cosine similarity, Pearson correlation, and Spearman correlation) were computed, and the criteria that at least two of these measures must be in agreement and at least one must be above 0.82 were applied. *Fcmr*^*−/−*^ cells that did not meet these criteria were labeled as “unassigned” to indicate that they did not correspond to any of the *Fcmr*^*+/+*^-exclusive clusters. Instead of using all genes to calculate the similarity, the method uses unsupervised feature selection to include only those genes that are most relevant for the underlying biological differences. We chose to use the top 1000 most relevant features as determined by this method.

To functionally characterize the cell subpopulations of interest, we carried out a pathway analysis using the GSVA R package^26^. Conceptually, GSVA is a non-parametric, unsupervised gene set enrichment (GSE) method that calculates sample-wise GSE scores as a function of genes inside and outside the input gene set. The methodology takes as input a normalized gene expression matrix with rows representing genes and columns representing samples or cells and a database of gene sets. The computations amount to a change in coordinate systems from genes to gene sets, resulting in an output scores matrix with rows as gene sets and columns retained as the original input samples. We subjected three different log-normalized expression matrices to this method, each with columns representing the *Fcmr*^*+/+*^ population paired with one of clusters 1 or 8 from the combined *Fcmr*^*+/+*^ and *Fcmr*^*−/−*^ t-SNE clustering, or the unassigned *Fcmr*^*−/−*^ subpopulation from the projection analysis. The rows of the matrices represented the 16,112 genes that remained after the QC step of the Seurat workflow. The gene sets used were the canonical pathways (c2.cp), and all gene ontology (GO) gene sets (c5) from the Broad Institute’s Molecular Signatures Database (MSigDB) v6.1^48^. The output matrices containing the GSVA pathway enrichment scores were then further used to perform differential pathway analysis between the *Fcmr*^*+/+*^ population and the subpopulations of interest (clusters 1, 8, and unassigned) using the limma R package^49^. This approach yielded the top differentially enriched pathways along with *p*-values adjusted for multiple testing correction using the Benjamini–Hochberg false discovery rate (FDR) controlling procedure^50^.

### Inguinal lymph node cell isolation

Mouse inguinal LNs were resected and placed into unsupplemented RPMI medium, diced using spring scissors, and enzymatically digested with Liberase TL (0.26 Wünsch U/ml; Roche) and DNase I (100 U/ml; Roche) in RPMI for 60 min at 37 °C. Dissociation was ensured by pipetting this mixture vigorously with a p1000 instrument every 10 min. After digestion, cells were strained through 40 μm nylon mesh, collected into 5 ml polystyrene tubes, and analyzed via flow cytometry.

### FITC migration assay

A 0.5% fluorescein isothiocyanate (FITC) solution was made by dissolving 50 mg FITC in 1:1 acetone:dibutylphthalate. Ventral–lateral abdominal hair of mice was trimmed using electric shears and stripped by application of hair removal solution (Nair©; Curch & Dwight), which was washed off the skin with soap and water. FITC solution (0.5%; 20 μl) was applied superior to the inguinal LN on the exposed skin and allowed to dry for 3–5 min. At 24 h after application of the FITC solution, inguinal LN were harvested and processed as described above and analyzed via flow cytometry.

### BMDC isolation and culture

BMDCs were induced to differentiate from stem/progenitor cells as previously described^51^. Briefly, total bone marrow cells were cultured in BMDC differentiation medium [RPMI (Gibco) supplemented with 10% FBS (Seradigm), 2 mM L-glutamine (Gibco), 100 IU penicillin, 100 μg/ml streptomycin (Gibco) (10% RPMI), and 40 ng/ml GM-CSF (PeproTech)]. BMDCs were harvested as the non-adherent fraction on day 10, washed twice in PRF HBSS, and resuspended at the appropriate cell density for further co-culture, stimulation, or biochemical analysis.

### BMDC-B16 cell co-culture

B16F0 cells were cultured and harvested as described above prior to treatment in vitro for 18 h with 0.25 µM staurosporine (Tocris Bioscience). Staurosporine-treated cells were washed, resuspended in RPMI containing 10% FBS, and co-cultured at a 2:1 ratio (2 × 10^6^ B16 cells:1 × 10^6^ BMDCs) in 100 mm Petri dishes. After 24 h, biological replicates were pooled and BMDCs were enriched using a CD11c^+^ selection kit (Miltenyi Biotec) according to the manufacturer’s instructions. Enriched BMDCs were plated in RPMI supplemented with 10% FBS in 96-well round bottom plates at a density of 1.5 × 10^5^ cells/well, and stimulated for 5 h with 50 ng/ml PMA plus 500 ng/ml ionomycin. Culture supernatants were collected and analyzed using the BioLegend LEGENDplex^TM^ mouse inflammation cytokine panel kit according to the manufacturer’s instructions. In every experiment, at least 4 technical replicates were analyzed for each sample.

### T cell proliferation assay

T cell proliferation was assessed using assays in which T cells were co-cultured with peptide-pulsed BMDCs. For the T cell component, *Pmel-1* CD8α^+^ T cells were isolated from spleens of naive mice to ≥95% purity using a negative selection kit (Miltenyi Biotec) according to the manufacturer’s instructions. Enriched CD8α^+^ T cells were resuspended at 5 × 10^6^ cells/ml in PBS containing 2.5 μM CTV (ThermoFisher) and incubated for 9 min at 37 °C. CTV was quenched with the addition of PRF HBSS containing 30% FBS. After centrifugation, cells were resuspended in RPMI containing 10% FBS to constitute CTV-labeled CD8α^+^ *Pmel-1* T cells.

For the BMDC component, BMDCs were isolated and cultured as described above prior to stimulation for 18 h with either 30 nM CpG (InvivoGen) or 10 μM 2′3′-cGAMP (InvivoGen). Stimulated BMDCs were washed twice with PBS, resuspended at 2 × 10^6^ cells/ml in PBS containing 10^−6^ M mgp100 peptide (25–33, EGSRNQDWL) (AnaSpec), and incubated at 37 °C for 30 min. Pulsed BMDCs were washed with PBS and resuspended in RPMI supplemented with 10% FBS to constitute stimulated peptide-pulsed BMDCs.

For co-cultures, CD8α^+^ *Pmel-1* T cells were cultured at a 3:1 ratio (120,000:40,000) with stimulated peptide-pulsed BMDCs in 96-well U-bottom tissue culture plates. Three biological replicates of BMDCs from *Fcmr*^+/+^ or *Fcmr*^*−*/*−*^ mice were analyzed in technical triplicates for each BMDC stimulus. At the time points indicated in the figure legend, cells were stained and processed for flow cytometry as described above, with modifications to volumes/concentrations where appropriate.

### DC vaccination

Naive C57Bl/6 mice received a ventrolateral intradermal injection of 2 × 10^5^ B16F10 cells that expressed the LCMV peptide gp33 (generously provided by Dr. Hanspeter Pircher^33^) and were resuspended in 50 μl PRF HBSS. On days 6 and 8 post-transplant, when tumors in mice had reached a palpable size, CpG-stimulated and peptide-pulsed BMDCs were intradermally injected (10^6^ cells in 100 µl) into the site contralateral to that of the tumor cell transplant. These BMDCs had been stimulated in vitro for 18 h with 30 nM CpG (ODN 1826, InvivoGen) and subsequently pulsed for 30 min at 37 °C with 10^−6^ M mgp100 peptide (25–33, EGSRNQDWL) and 10^−6^ M LCMV-derived gp33 peptide (33–41, KAVYNFATM) (AnaSpec) in PBS. Stimulated peptide-pulsed BMDCs were washed and resuspended at 10^7^ cells/ml in PRF HBSS in preparation for injection.

After BMDC injection, the sizes of developing tumors were measured with a digital caliper every 3rd day. Measurements were performed blind to genotype of the donor BMDCs. Tumor volume was calculated as [(short side)^2^ × (long side)]/2.

For depletion of CD8α^+^ T cells, the anti-CD8α monoclonal antibody (mAb) clone YTS169 was intraperitoneally (IP) injected (100 μg in 100 μl). Two injections were delivered to each individual mouse. One mAb injection was delivered 6 days post DC vaccination, and one injection 8 days post DC vaccination. The sizes of developing tumors were measured over the course of the experiment with a digital caliper. Measurements were performed blind to genotype of the donor BMDCs and mAb treatment. Tumor volume was calculated as [(short side)^2^ × (long side)]/2.

### Immunoblotting

BMDCs (4 × 10^6^) isolated, differentiated and cultured as described above were aliquoted into 250 μl PRF HBSS in 1.5 ml screw-cap polypropylene tubes. Cells were starved for 2 h at 37 °C prior to stimulation by the addition of 250 μl 2×-concentrated stimuli at the appropriate time points. Stimulation was stopped with the addition of 1 ml ice-cold HBSS and transfer to wet ice. Cells were immediately centrifuged at 8000*g* for 1 min at 4 °C, snap-frozen on dry ice or liquid N_2_, and stored at −80 °C until analysis.

Cells were lysed using RIPA buffer (50 mM Tris–HCl pH 7.4, 1% NP-40, 0.25% Na-deoxycholate, 150 mM NaCl, 1 mM EDTA). Lysates were clarified by centrifugation at 10,000*g* for 10 min and the supernatants transferred to new 1.5 ml tubes. Protein contents of the lysates were quantified using the BCA 96-well plate assay (ThermoFisher) according to the manufacturer’s instructions. Samples were fractionated on 4–12% gradient NuPAGE Bis–Tris precast polyacrylamide gels, followed by transfer to polyvinylidene difluoride (PVDF) membranes according to the manufacturer’s instructions (Invitrogen; ThermoFisher). Blots were probed with anti-phospho-NFκB p65 (Ser536), anti-vinculin, anti-β-actin (Cell Signaling), and anti-NFκB p65 (Santa Cruz) antibodies. Uncropped immunoblot scans are presented in Supplementary Fig. 9.

### Generation of Toso-Fc therapeutic

The Toso-Fc therapeutic used was comprised of the mouse Fcmr extracellular (EC) domain linked to mouse IgG Fc. The mouse Toso-Fc (mTosoFc) was produced as previously described for the human Toso-Fc^13^. Specifically, the sense primer 5′-GAATTCGAGAGTCCTCCCAGAAGTACAGCTGAATG-3′ and antisense primer 5′-AGATCTAAATTCTGGGATGGGGATGTGAAGC-3′ were used to PCR amplify a 751 base pair fragment spanning the extracellular region of mouse Fcmr (Toso/Faim3). This fragment was sequence verified and ten subcloned in-frame downstream of the human IL2 signal sequence and upstream of mouse FC regions using the commercially available mammalian expression vector pFUSE-IL2SS-mouseIgGAe1-Fc2 (InvivoGen).

The mToso-Fc was expressed in 293FT cells and purified from cell-culture supernatants using protein-G sepharose (Sigma) affinity chromatography and FPLC (Pharmacia). Purity was assessed through PAGE and size exclusion FPLC. In addition, endotoxin contamination was assessed with a commercially available kit (GenScript). Multiple batches of purified protein were assessed, and endotoxin was determined to be ≤0.08 EU/ml.

### Statistical methods

Where applicable, differences between groups were assessed using ANOVA or two-tailed *t* tests. Predictive relationships between variables were evaluated using linear regression analyses. All statistical analyses were performed using Prism software (GraphPad). In all panels, data are presented as mean ± SEM. Differences with *p* values ≤ 0.05 (CI ≥ 0.95) were considered statistically significant.

### Reporting summary

Further information on research design is available in the Nature Research Reporting Summary linked to this article.

## Supplementary Information

Supplementary Information

Reporting Summary

## Data Availability

The authors declare that all data supporting the findings of this study are available within this article and its Supplementary Information files, or from the corresponding author upon reasonable request. Single cell RNA-sequencing data have been deposited in the Gene Expression Omnibus database under the accession code GSE130287.
