# Author Correction: Fcmr regulates mononuclear phagocyte control of anti-tumor immunity

**DOI:** 10.1038/s41467-019-12106-8

**Published:** 2019-09-03

**Authors:** Shawn P. Kubli, Larsen Vornholz, Gordon Duncan, Wenjing Zhou, Parameswaran Ramachandran, Jerome Fortin, Maureen Cox, SeongJun Han, Robert Nechanitzky, Duygu Nechanitzky, Bryan E. Snow, Lisa Jones, Wanda Y. Li, Jillian Haight, Andrew Wakeham, Mark R. Bray, Tak W. Mak

**Affiliations:** 1 The Campbell Family Institute for Breast Cancer Research, Princess Margaret Cancer Centre, 610 University Avenue, Toronto, ON M5G 2M9 Canada; 2 Department of Medical Biophysics, University of Toronto, 101 College Street, Toronto, ON M5G 1L7 Canada; 3 Department of Immunology, University of Toronto, 101 College Street, Toronto, ON M5G 1L7 Canada; 4 Department of Medicine, University of Hong Kong, Pok Fu Lam, 999077 Hong Kong

**Keywords:** Immunology, Immunotherapy, Innate immune cells, Tumour immunology

Correction to: *Nature Communications* 10.1038/s41467-019-10619-w, published online 18 June 2019.

The original version of this Article contained an error in the Data availability statement. The accession code indicated ‘GSM130287’ and should have read ‘GSE130287’. This has been corrected in both PDF and HTML versions of the Article.

