# Supplementary material for: Fcmr regulates mononuclear phagocyte control of anti-tumor immunity
Source: Nat Commun. 2019 Jun 18;10:2678. doi: 10.1038/s41467-019-10619-w (PMC6581943; doi:10.1038/s41467-019-10619-w)
Supplement: Supplementary file 1 — Supplementary Information [file 41467_2019_10619_MOESM1_ESM.pdf]

Fcmr regulates mononuclear phagocyte control of anti-tumor immunity

Kubli *et al.*

Supplementary Information

Supplementary Figure 1

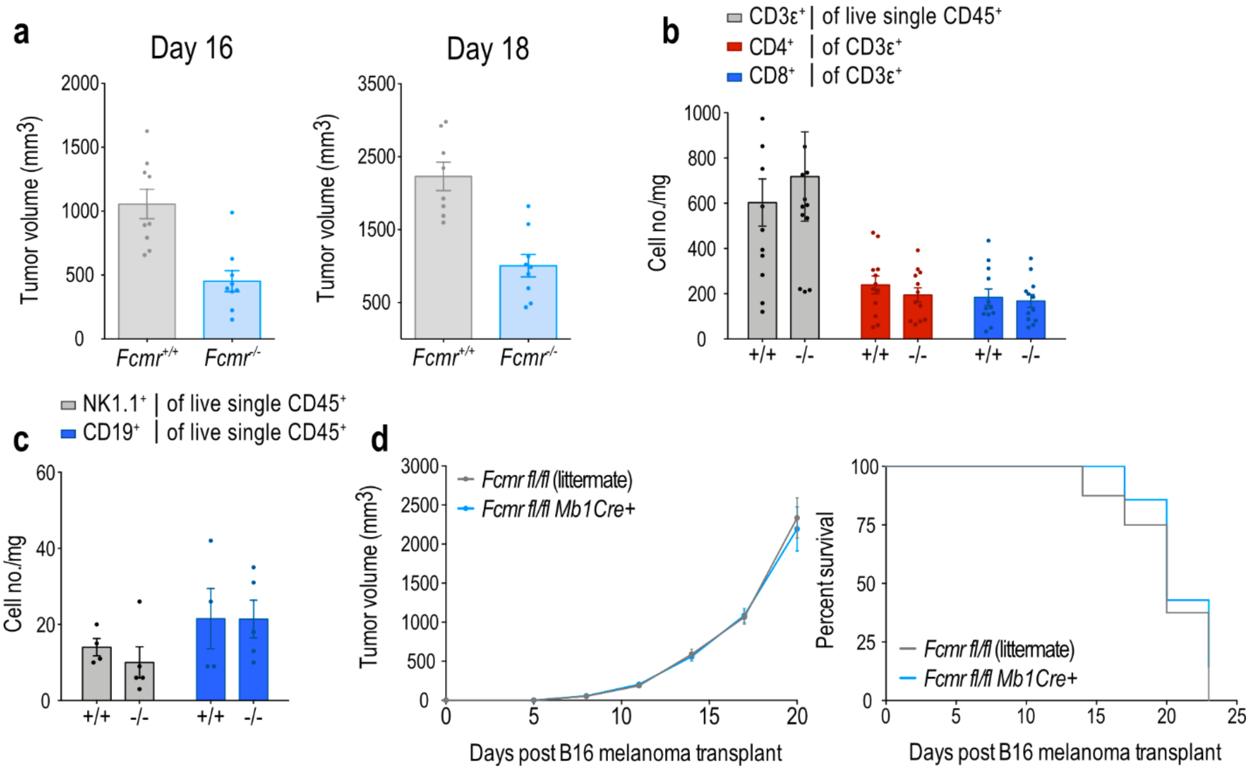

Supplementary Figure 1. Fcmr inhibits myeloid cell-dependent anti-tumor immunity

a Quantification of tumor volumes for *Fcmr*$^{+/+}$ and *Fcmr*$^{-/-}$ mice at 16 days (left) and 18 days (right) after ventral-lateral intradermal transplant of B16F0 cells (2x10^{5}) superior to the inguinal lymph node (LN). Data points represent individual mice (n=9 *Fcmr*$^{+/+}$ and 8 *Fcmr*$^{-/-}$ mice).

b, c Quantification of tumor-infiltrating cells in B16F0 tumors harvested from the *Fcmr*$^{+/+}$ and *Fcmr*$^{-/-}$ mice in **a**. Cell densities of **b** the total T cell population (CD3ε^{+}) and CD4^{+} and CD8^{+} T cell subpopulations, and **c** the B and NK cell populations, in harvested tumors are shown. All cell density measurements were normalized to tumor mass. Each data point represents an individual mouse.

d Time course of (left) B16F0 tumor growth and (right) mouse survival for*Fcmr*$^{fl/fl}$;*Mb1Cre*$^{+}$ and *Fcmr*$^{fl/fl}$ littermate mice (n=8 *Fcmr*$^{fl/fl}$ and 7 *Fcmr*$^{fl/fl}$; *Mb1Cre*$^{+}$ mice) that received transplant of B16F0 cells as in **a**.

Data are represented as mean ± SEM (ANOVA, *t* test; *p<0.05; **p<0.01; ***p<0.001)

Supplementary Figure 2

a

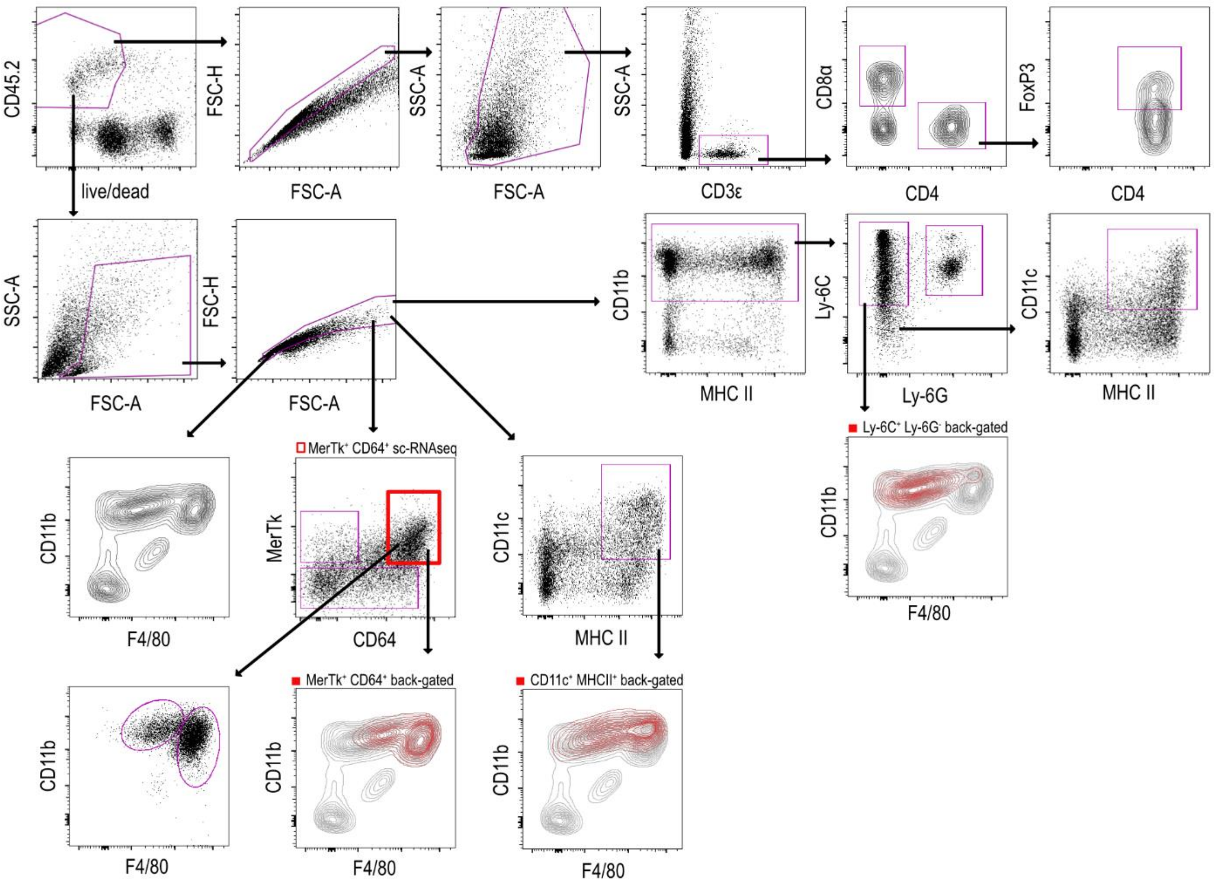

b

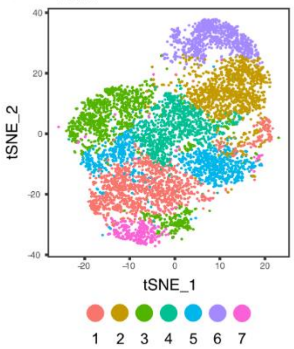

c

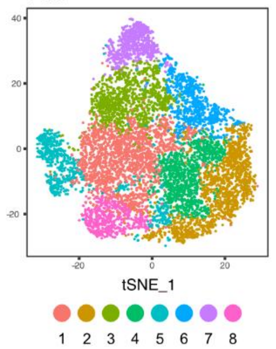

d

Fcmr$^{+/+}$ **only cluster**	**No. of Fcmr**$^{-/-}$ **cells**
1	1273
2	916
3	765
4	691
5	1662
6	756
7	639
**unassigned**	1297

e

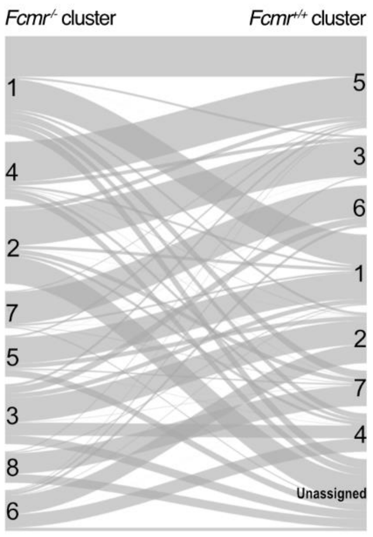

f

**Transcription Factor**	*****logFC**
Irf7	0.633
Stat1	0.574
Basp1	0.336
Mxi1	0.285
Polr2l	0.282
Hif1a	0.281
Ugp2	0.257
Klf2	-0.256
Fosb	-0.272
Egr1	-0.427

Supplementary Figure 2. Fcmr deficiency alters the functional heterogeneity of TMPs

a Representative flow cytometry plots illustrating the gating schemes used to quantify tumor-infiltrating immune cells in the B16F0 tumors from the *Fcmr*$^{+/+}$ and *Fcmr*$^{-/-}$ mice in Supplemental Figure 1a.

b, c t-SNE representation of cell clusters from FACS-sorted TMPs isolated from the B16F0 tumors of the *Fcmr*$^{+/+}$ and *Fcmr*$^{-/-}$ mice in Figure S1A. *Fcmr*$^{+/+}$ cells **b** were clustered separately from *Fcmr*$^{-/-}$ cells **c**.

d Table illustrating the number of *Fcmr*$^{-/-}$ cells that could be "assigned" to each of the 7 identified *Fcmr*$^{+/+}$ cell clusters in **b**, and those *Fcmr*$^{-/-}$ cells that could not be assigned (unassigned).

e Sankey diagram showing the proportion of *Fcmr*$^{-/-}$ cells from each representative cluster in the analysis of *Fcmr*$^{-/-}$ cells in **c** that were "assigned" to each of the 7 identified *Fcmr*$^{+/+}$ cell clusters in **b**. Unassigned *Fcmr*$^{-/-}$ cells are also shown.

f Table illustrating the significantly differentially expressed transcription factors for unassigned *Fcmr*$^{-/-}$ cells and the associated log fold change (logFC) expression difference compared to all Fcmr$^{+/+}$ cells.

Supplementary Table 1

**Pathway**	**logFC**	**t**	**B**
Endosomal vacuolar pathway	0.37	23.1	249.2
Antigen processing and presentation of endogenous peptide antigen	0.28	23.1	249.9
Antigen processing and presentation of endogenous antigen	0.23	20.8	202.6
MHC protein complex	0.19	17.7	145.1
MHC class I protein complex	0.14	12.6	70.2
Type I interferon receptor binding	0.20	32.8	494.0
Type I interferon pathway	0.20	19.3	174.1
Interferon-α and -β signaling	0.14	18.6	161.0
Response to type I interferon	0.16	20.2	191.8
Interferon-γ production	0.12	15.3	107.8
Interferon-γ pathway	0.17	18.2	153.5
Interferon-γ signaling	0.10	15.1	104.8
Negative regulation of IL-10 production	0.19	26.9	338.4
Positive regulation of cytokine production	0.15	23.0	248.5
Cytotoxic T lymphocyte pathway	0.22	28.5	377.6
Positive regulation of T cell mediated cytotoxicity	0.15	18.5	159.8
Positive regulation of T cell cytokine production	0.14	18.1	152.6
T cytotoxic pathway	0.15	18.2	153.8
Regulation of T cell mediated cytotoxicity	0.12	18.1	152.0
Positive regulation of αβ T cell proliferation	0.11	15.9	116.9

Pathways indicated are those found enriched in unassigned *Fcmr*$^{-/-}$ tumor-associated mononuclear phagocytes (MPs) as compared to all *Fcmr*$^{+/+}$ MPs. Values illustrated are the log2-fold-changes of the linearly modeled GSVA scores of the two groups being compared, along with the moderated t-statistic (t) and the B-statistic (the log-odds that a pathway is differentially expressed). Adjusted P-values (using the Benjamini-Hochberg procedure[45]) for all the pathways indicated are $P \leq 1 \times 10^{-35}$.

Supplmentary Table 2

Pathway	logFC	t	B
Creation of C4 and C2 activators	-0.15	-9.8	39.4
Gap junction trafficking	-0.15	-17.1	135.3
Sterol catabolic process	-0.12	-14.4	94.8
Regulation of amyloid precursor protein catabolic process	-0.12	-15.8	114.8
Negative regulation of dendritic spine development	-0.11	-14.0	89.3
Regulation of cytokine secretion involved in immune response	-0.10	-12.6	71.5
Positive regulation of insulin-like growth factor receptor signaling	-0.10	-10.7	49.3
S1P and S1P4 pathway	-0.08	-11.6	59.2
Vitamin D metabolic process	-0.08	-8.5	28.4
Gamma aminobutyric acid signaling	-0.08	-10.9	51.5
Positive regulation of cardiac muscle cell differentiation	-0.08	-10.4	46.3
Positive regulation of response to oxidative stress	-0.07	-11.9	62.8
PLCE pathway	-0.07	-9.8	39.8
HDL mediated lipid transport	-0.07	-8.9	31.8
Positive regulation of macrophage differentiation	-0.07	-10.2	43.9
Clathrin binding	-0.07	-15.3	106.7
Tryptophan metabolism	-0.06	-12.9	74.4
Cellular modified amino acid catabolic process	-0.06	-9.8	39.7
Neutral lipid catabolic process	-0.06	-9.2	33.9
Positive regulation of non-canonical Wnt signaling pathway	-0.05	-8.1	24.6

Pathways indicated are those found relatively downregulated in unassigned *Fcmr*$^{-/-}$ tumor-associated mononuclear phagocytes (MPs) as compared to all *Fcmr*$^{+/+}$ MPs. Values illustrated are the log2-fold-changes of the linearly modeled GSVA scores of the two groups being compared, along with the moderated t-statistic (t) and the B-statistic (the log-odds that a pathway is differentially expressed). Adjusted P-values (using the Benjamini-Hochberg procedure[45]) for all the pathways indicated are $P \leq 1 \times 10^{-35}$.

Supplementary Figure 3

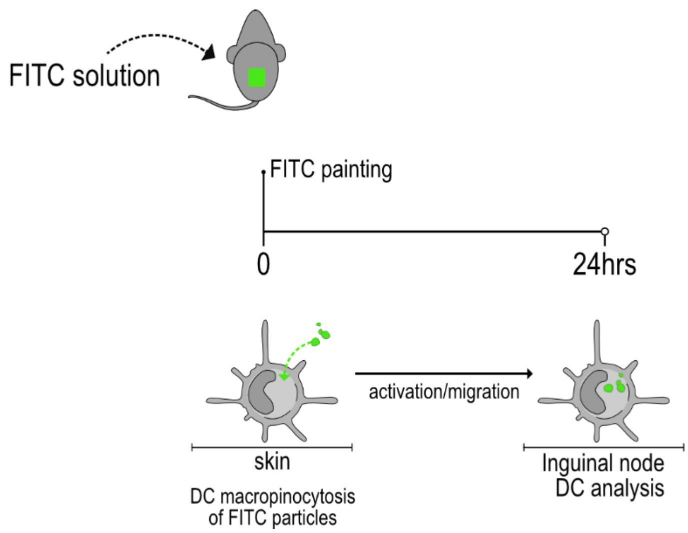

Supplementary Figure 3. Fcmr inhibits DC phagocytosis, maturation, and migration

Schematic diagram of the experiment showing the ventral-lateral application of FITC solution (FITC-dibutylthalate) (top), the timeline of the experiment (middle), the uptake of FITC by skin DCs (bottom left), and DC trafficking to the inguinal lymph node followed by analysis (bottom right).

Supplementary Figure 4

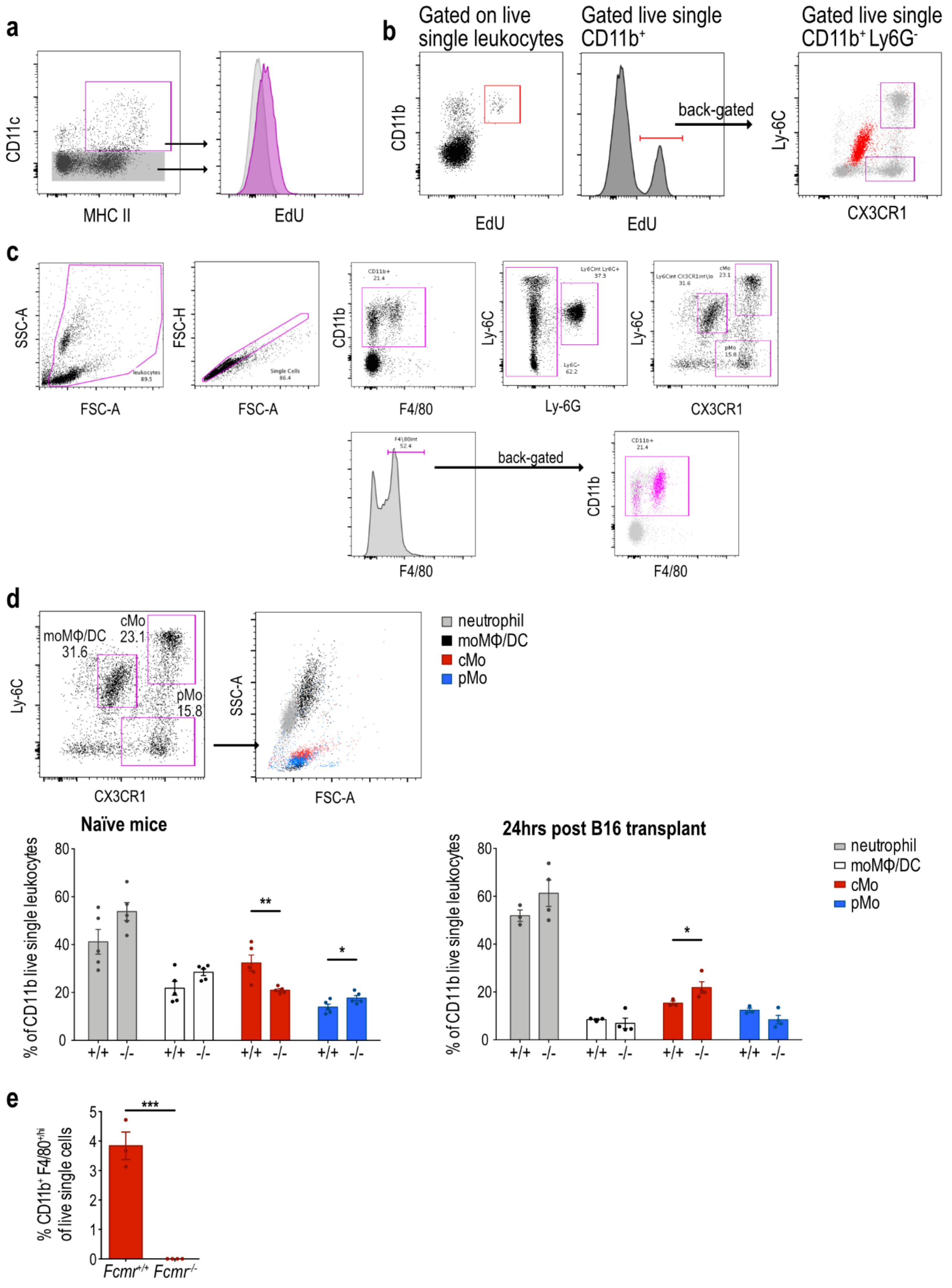

Supplementary Figure 4. Fcmr inhibits MP uptake of cancer cell DNA

a Left: Representative flow cytometry plot illustrating gating strategy identifying total inguinal LN DCs ($CD11c^+$ MHC II^+) and lymphocytes ($CD11c^-$ MHC $II^{+/-}$). Right: Representative histogram showing EdU-positive DCs (purple) and EdU-negative lymphocytes (grey).

b Left: Representative flow cytometry plot illustrating EdU-positive and -negative leukocyte populations in the spleens of *Fcmr*$^{+/+}$ mice (n=5) at 24 hrs after receiving ventral-lateral intradermal injection of EdU-labeled B16 melanoma cells (B16.EdU). Middle: Representative histogram of EdU-positive and -negative myeloid cells in the mice in the left panel. Right: Back-gating of the EdU^+ population (red) among the cells in the middle panel.

c Representative flow cytometry plot illustrating the gating strategy for the identification of the various myeloid cell populations in peripheral blood (PB).

d Top left: Representative flow cytometry plot illustrating three MP subpopulations in mouse PB. Top right: Back-gating to show forward and side-scatter properties of the cell subsets in the top left panel. Bottom: Quantification of the populations in the top left panel in naïve *Fcmr*$^{+/+}$ and *Fcmr*$^{-/-}$ mice (left), and in *Fcmr*$^{+/+}$ and *Fcmr*$^{-/-}$ mice at 24 hrs post-intradermal transplant of B16 cells (right). Data points represent individual mice.

e Quantification of $CD11b^{hi}$ $F4/80^{hi}$ MPs in PB of *Fcmr*$^{+/+}$ and *Fcmr*$^{-/-}$ mice at 24 hrs post-intradermal B16 cell transplant.

Data are represented as mean ± SEM (*t* test; *p<0.05; **p<0.01; ***p<0.001)

Supplementary Figure 5

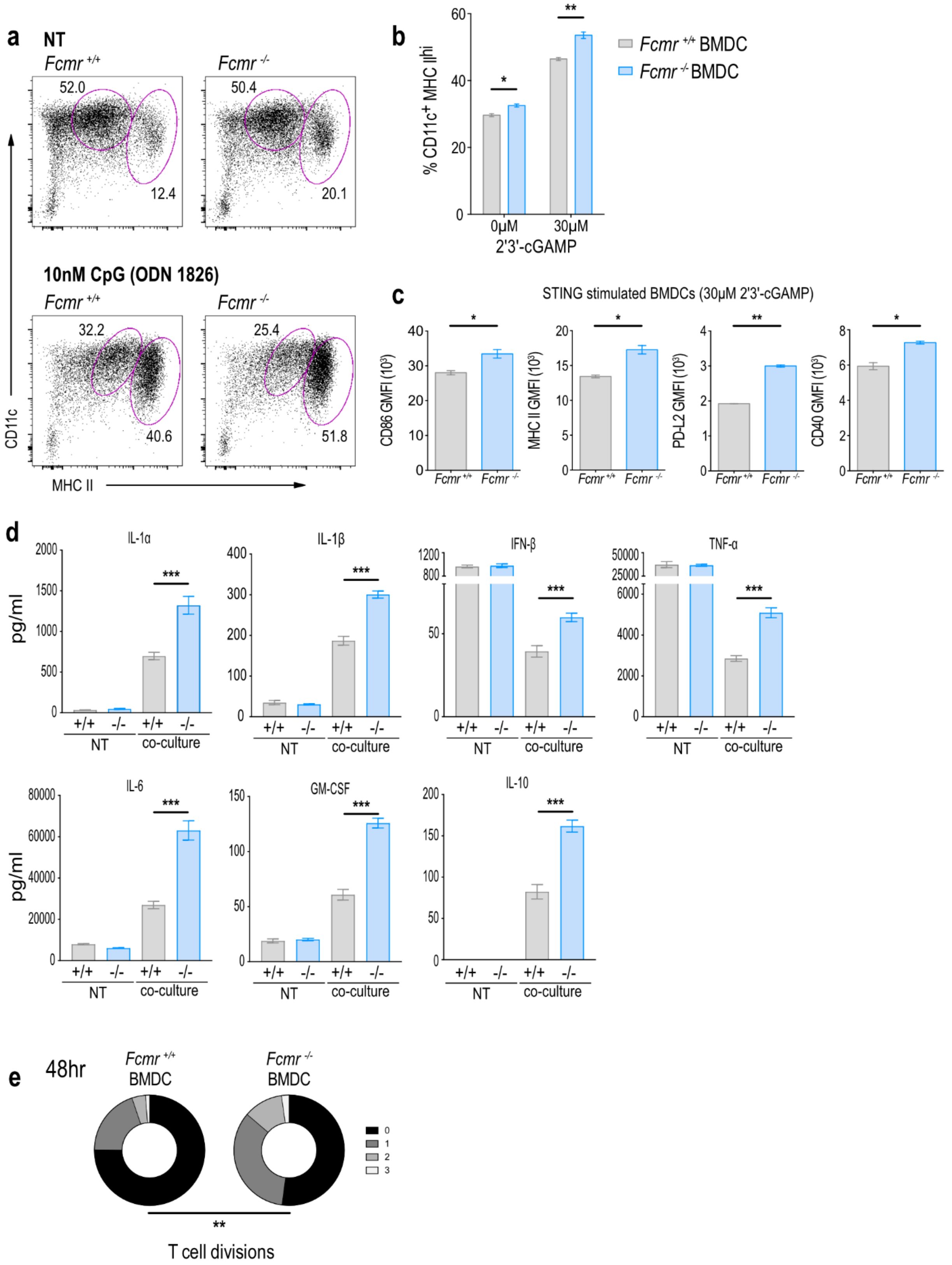

Supplementary Figure 5. Fcmr inhibits BMDC maturation

a Representative flow cytometry plots illustrating the gating strategy to identify immature ($CD11c^{+}$ MHC II^{int}) and mature ($CD11c^{+}$ MHC II^{hi}) BMDCs from *Fcmr*$^{+/+}$ (left) and *Fcmr*$^{-/-}$ (right) littermate mice (n=3/group) that were not treated (NT) (top), or stimulated with 10 nM CpG (bottom).

b Quantification of mature ($CD11c^{+}$ MHC II^{hi}) *Fcmr*$^{+/+}$ and *Fcmr*$^{-/-}$ BMDCs following stimulation of the cyFcmrlic DNA-sensing pathway with the STING agonist 2’3’-cGAMP. Data represent 3 biological replicates.

c Quantification of GMFI values for the indicated co-stimulatory molecules expressed by the stimulated BMDCs in **b**.

d Quantification of the indicated cytokines produced by *Fcmr*$^{+/+}$ and *Fcmr*$^{-/-}$ BMDCs under NT or co-culture conditions. Data are pooled from three biological replicates.

e Quantification of T cell divisions at 48 hrs after co-culture with the *Fcmr*$^{+/+}$ and *Fcmr*$^{-/-}$ BMDCs. Data are from 3 technical replicates within each of 3 biological replicates.

Data are represented as mean ± SEM (ANOVA, *t* test; *p<0.05; **p<0.01; ***p<0.001)

Supplementary Figure 6

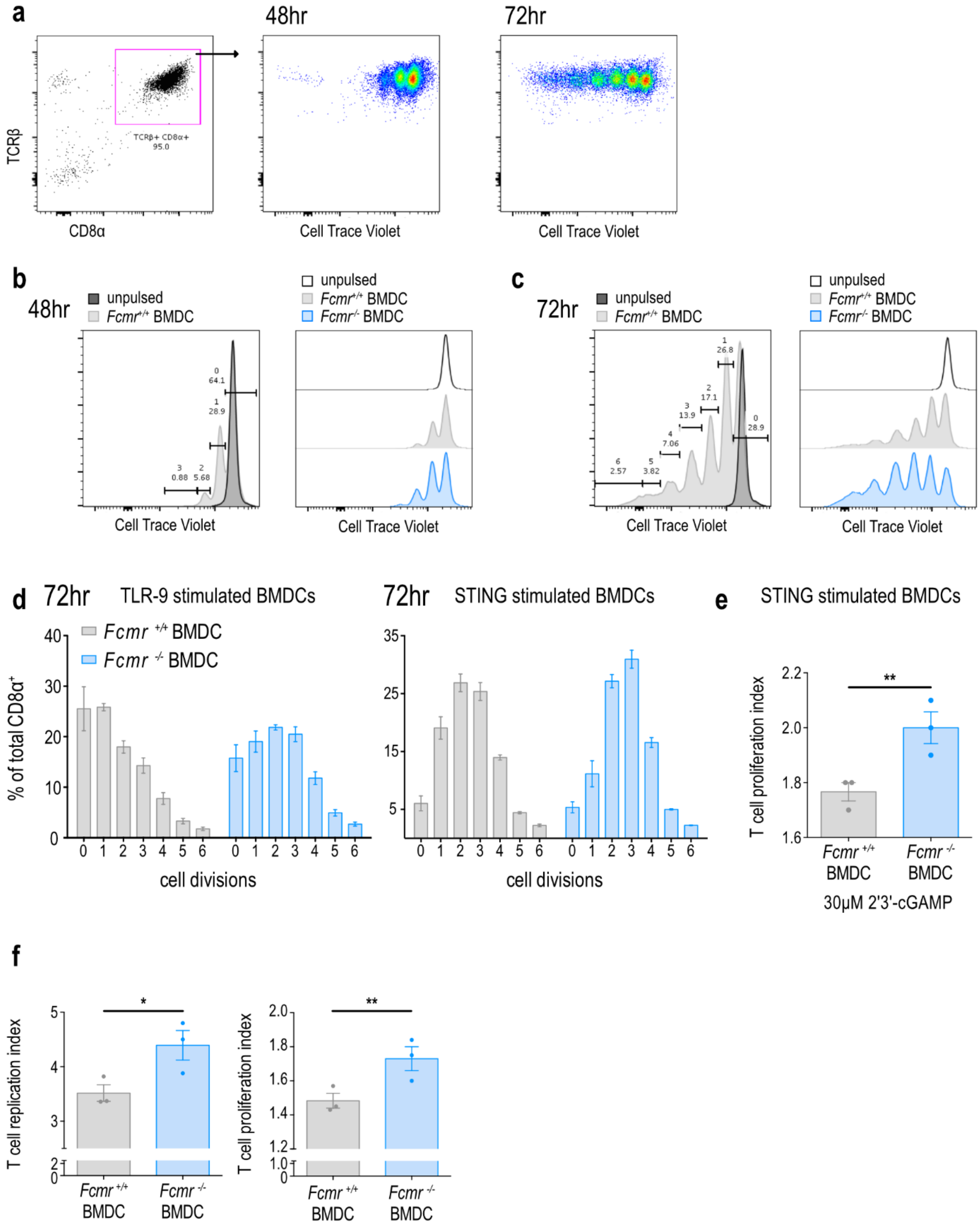

Supplementary Figure 6. Fcmr inhibits BMDC-dependent T cell activation

a Representative flow cytometry plots illustrating the proliferation of Pmel TCR transgenic mouse $CD8\alpha^+$ T cells after co-culture with BMDCs that had been stimulated and peptide-pulsed as described in Figure 6. Left: Cells were gated on $TCR\beta^+$ $CD8\alpha^+$. Right: Cell Trace Violet dot plots at 48 and 72 hrs after co-culture initiation.

b, c Left: Representative histogram illustrating the gating strategy to track the number of divisions of CVT-labelled T cells after co-culture with TLR9-activated BMDCs that were pulsed with peptide (or not). Right: Representative histograms comparing T cell divisions at **b** 48 hrs or **c** 72 hrs after co-culture with either activated unpulsed BMDCs, activated pulsed *Fcmr*$^{+/+}$ BMDCs, or activated pulsed *Fcmr*$^{-/-}$ BMDCs.

d Quantification of the percentage of $CD8\alpha^+$ T cells that divided 0 to 6 times after 72 hrs of co-culture with *Fcmr*$^{+/+}$ or *Fcmr*$^{-/-}$ BMDCs that had been stimulated with the TLR-9 agonist CpG (left) and STING agonist 2’3’cGAMP (right). Data are from 3 technical replicates within each of 3 biological replicates.

e T cell proliferation index in the co-culture with *Fcmr*$^{+/+}$ or *Fcmr*$^{-/-}$ BMDCs that had been stimulated with STING agonist 2’3’cGAMP. Data are from 3 technical replicates within each of 3 biological replicates. Each data point represents a biological replicate.

f T cell replication index (left) and proliferation index (right) in the co-culture with *Fcmr*$^{+/+}$ or *Fcmr*$^{-/-}$ BMDCs that had been stimulated with TLR-9 agonist CpG. Data are from 3 technical replicates within each of 3 biological replicates. Each data point represents a biological replicate.

Data are represented as mean ± SEM (ANOVA, *t* test; *p<0.05; **p<0.01; ***p<0.001)

Supplementary Figure 7

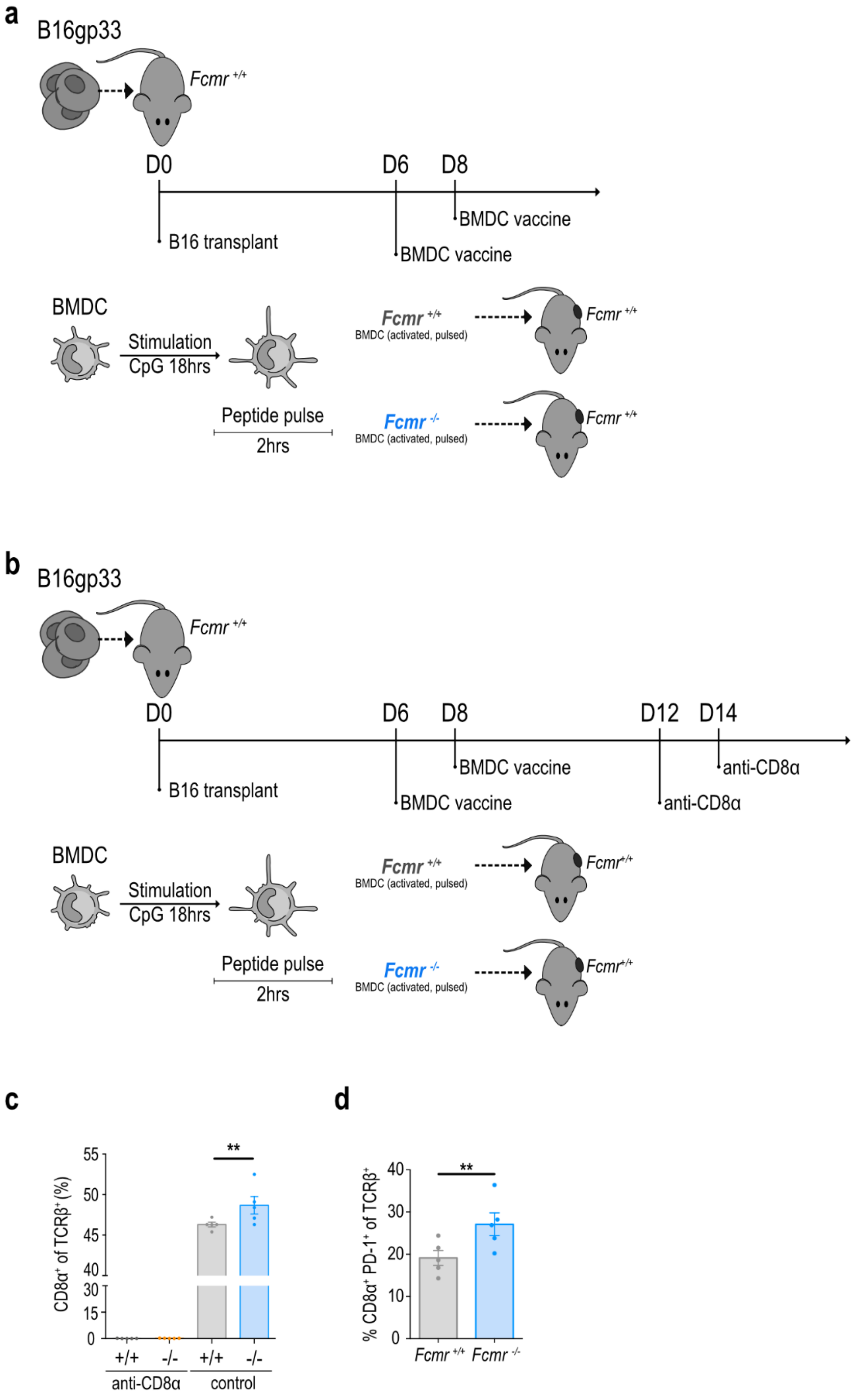

Supplementary Figure 7. Fcmr inhibits DC-dependent T cell anti-tumor immunity

a, b Top: Schematic diagram of the experiment illustrating the ventral-lateral transplantation of B16gp33 cells (B16 cells ectopically expressing the LCMV peptide gp33) into *Fcmr*$^{+/+}$ recipient mice via intradermal injection superior to the inguinal LN. *Fcmr*$^{+/+}$ and *Fcmr*$^{-/-}$ BMDC vaccines were administered on days 6 and 8 post-transplant. Bottom: To make BMDC vaccines, *Fcmr*$^{+/+}$ and *Fcmr*$^{-/-}$ BMDCs were stimulated for 18 hrs with CpG, pulsed with gp33 peptide for 2 hrs, and transferred to B16gp33 tumor-bearing *Fcmr*$^{+/+}$ mice via intradermal injection contralateral to the tumor site.

b Schematic diagram representing experimental design as for **b** above, with the addition of anti-CD8α antibody treatment on days 12 and 14 post-transplant.

c Quantification of the percent CD8α^{+} of TCRβ^{+} T cells in the peripheral blood of DC vaccinated mice from **b**, with either anti-CD8α monoclonal antibody treatment or control.

d Quantification of the percent CD8α^{+} PD-1^{+} of TCRβ^{+} T cells in the peripheral blood of *Fcmr*$^{+/+}$ and *Fcmr*$^{-/-}$ DC vaccinated control mice from **b**.

Data are represented as mean ± SEM (*t* test; *p<0.05; **p<0.01; ***p<0.001)

Supplementary Figure 8

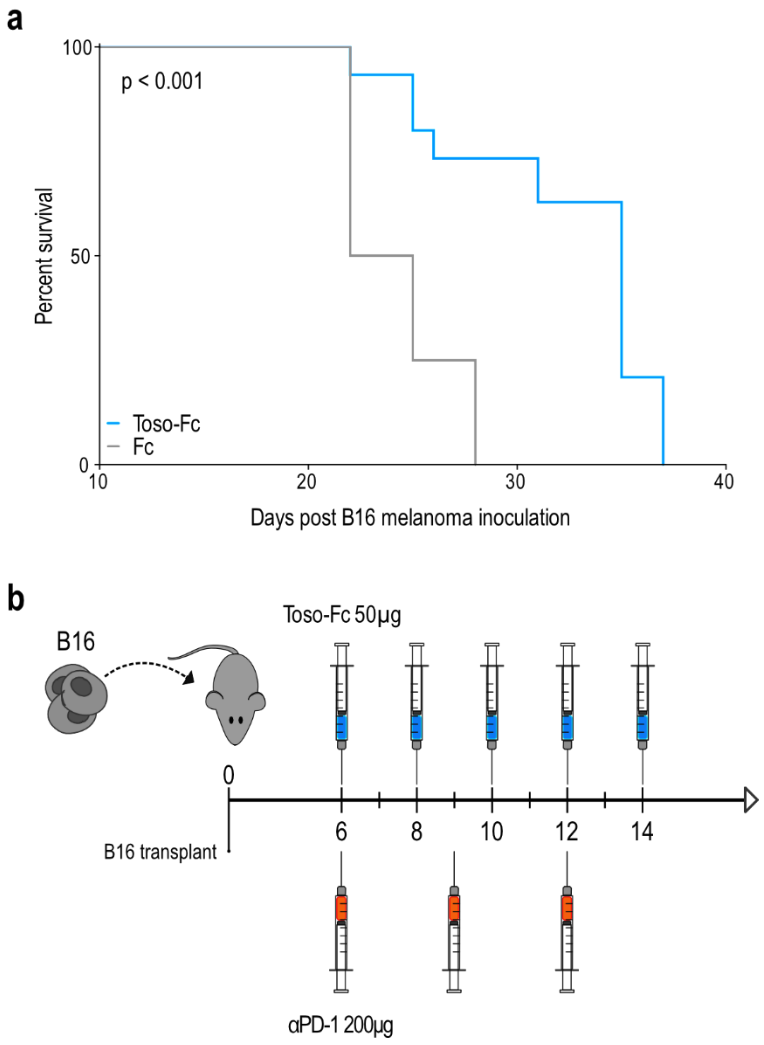

Supplementary Figure 8. Toso-Fc increases survival and synergizes with anti-PD-1

a Survival curve for the mice in Figure 6a.

b Schematic diagram of the experiment showing the ventral-lateral transplantation of B16F0 cells superior to the inguinal LN via intradermal injection into *Fcmr*$^{+/+}$ recipient mice (left), and the associated timeline of treatment with Toso-Fc fusion protein decoy receptor and anti-PD-1 antibody starting 6 days post-transplant (right).

Supplementary Figure 9

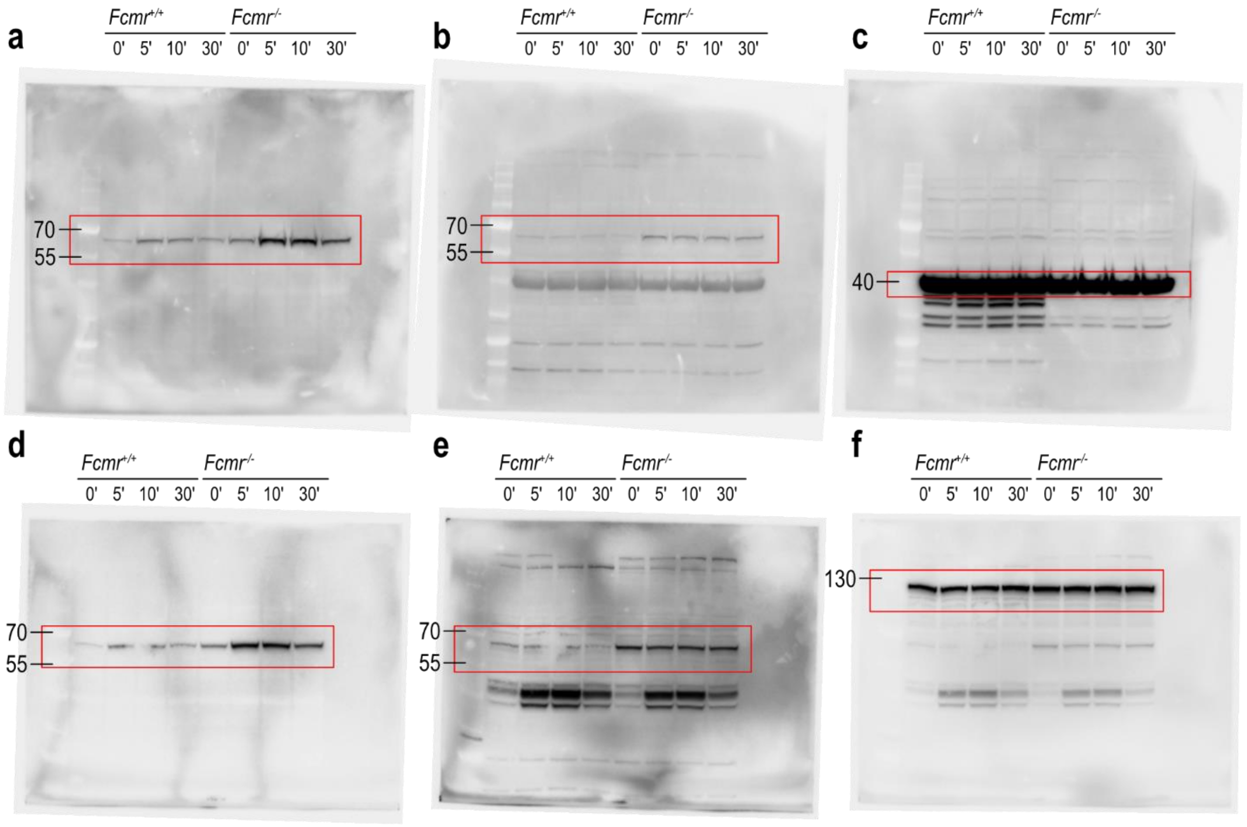

Supplementary Figure 9. Total p65 and phospho-p65 in LPS stimulated BMDCs

Immunoblot analysis of total p65 and phospho-p65 protein levels in *Fcmr*$^{+/+}$ and *Fcmr*$^{-/-}$ BMDCs that were stimulated with 10 µg/ml LPS (to engage TLR-4) for the indicated times. Two representative uncropped immunoblots are shown; one with β-actin loading control **c**, and one with vinculin loading control **f**. Data are representative of three separate biological replicates.

a-c Uncropped immunoblot from Figure 5c that was probed with anti-phospho-p65 **a**, stripped and re-probed for total p65 **b**, and β-actin **c**.

d-f Uncropped immunoblot with a different loading control, vinculin, representing an additional biological replicate. The blot was probed with anti-phospho-p65 **d**, stripped and re-probed for total p65 **e**, and vinculin **f**.
